# Glycyrrhetinic Acid-Functionalized Mesoporous Silica Nanoparticles for the Co-Delivery of DOX/CPT-PEG for Targeting HepG2 Cells

**DOI:** 10.3390/pharmaceutics12111048

**Published:** 2020-11-02

**Authors:** Gabriel Martínez-Edo, Cristina Fornaguera, Salvador Borrós, David Sánchez-García

**Affiliations:** Grup d’Enginyeria de Materials (GEMAT), Institut Químic de Sarrià (IQS), Universitat Ramon Llull (URL), Via Augusta, 390, 08017 Barcelona, Spain; gabrielmartineze@iqs.edu (G.M.-E.); cristina.fornaguera@iqs.url.edu (C.F.); salvador.borros@iqs.url.edu (S.B.)

**Keywords:** mesoporous silica nanoparticles, dual release, doxorubicin, camptothecin, anticancer drugs, combination therapy, targeting systems

## Abstract

A pH-triggered mesoporous silica nanoparticle (MSN)-based nano-vehicle for the dual delivery of doxorubicin (DOX)/camptothecin-PEG (CPT-PEG) has been prepared. To enhance its selectivity, the nanoparticles were decorated with glycyrrhetinic acid (GA) to target HepG2 cells. The highly insoluble CPT was derivatized with a reductive-cleavable PEG chain to improve its loading within the MSN. The preparation of these particles consisted of four steps. First, CPT-PEG was loaded within the pores of the MSN. Then, dihydrazide polyethylene glycol chains were introduced onto the surface of an aldehyde-functionalized MSN by means of a hydrazone bond. Afterwards, DOX was covalently attached to the other end of the dihydrazide polyethylene glycol chains. Finally, the resulting nanoparticles were decorated with GA by formation of an imine bond between the amino group of DOX and a benzaldehyde-GA derivative. The system was stable at physiological conditions and the release of both drugs was negligible. However, at acidic pH, a burst release of DOX and a gradual release of CPT-PEG takes place. GA-decorated drug delivery systems (DDS) selectively internalizes into HepG2. In vitro tests demonstrated that this system shows a great cytotoxicity towards HepG2 cells. Furthermore, glutathione cleavage of CPT prodrug assures the formation of free CPT leading to a synergistic effect in combination with DOX.

## 1. Introduction

Hepatocellular carcinoma has become one of the world’s most devastating diseases because of its high morbidity and high mortality [1]. Up to now, liver transplantation is the preferred choice applied as a treatment, but its application is usually hampered due to the lack of donors. An alternative treatment is the surgical intervention, but it is often too complex due to the inaccessibility of the tumors [2,3]. Therefore, chemotherapy has become the standard of care to fight this malignancy. Nevertheless, the major issue associated with the administration of chemotherapeutics is their high toxicity and lack of selectivity leading to systemic toxicity that can be detrimental for the patient quality of life [4].

To address these drawbacks, drug delivery systems (DDS) based on nanoparticles (NP) have been proposed. In particular, in the last years, mesoporous silica nanoparticles (MSN) have emerged as effective platforms to prepare smart nano-carriers to ferry drugs with precision to the tumors owing to its outstanding properties such as good biocompatibility, easy chemical modification, and high encapsulation capacity [5,6,7,8,9,10,11,12,13,14,15,16]. These DDSs accumulate selectively into the tumors thanks to the so-called enhanced permeability retention effect (EPR effect) [13,17]. This selectivity can be improved with the installation of ligands on the surface of the NP able to recognize and bind to receptors overexpressed by tumor cells [4,13,18,19]. It has been reported that human hepatocellular carcinoma HepG2 cells possess a significant number of glycyrrhetinic acid (GA) and hyaluronic acid (HA) receptors in its membrane [3,4,17,20]. This feature has been capitalized on the design of selective DDSs and the surface of nanoparticles has been decorated with these ligands to enhance the concentration of the nanocarrier in the tumoral tissue [4,20,21,22].

**Figure 1:**
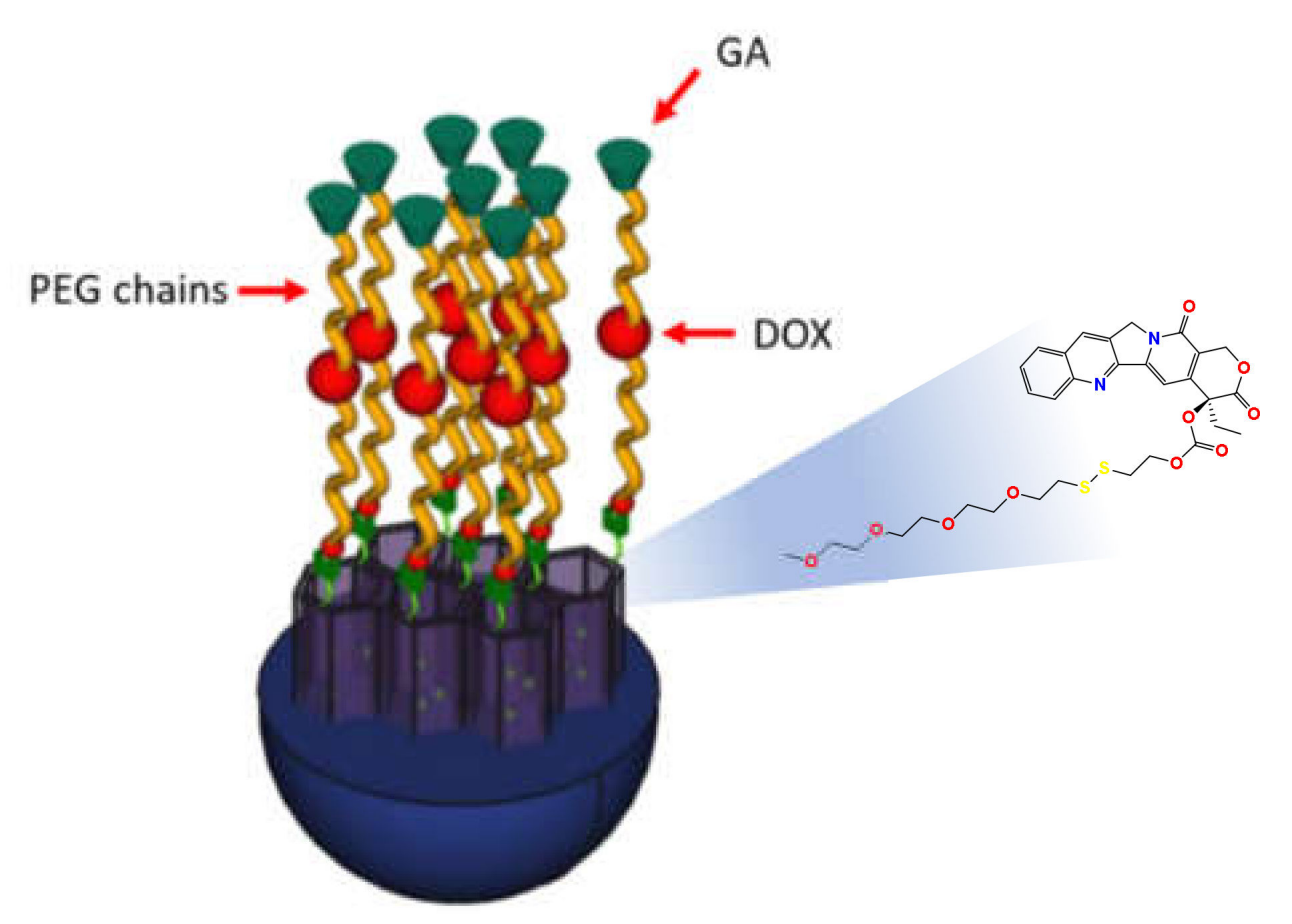
Schematic representation of the drug delivery systems (DDS).

In this work, it is proposed to selectively vehiculize two chemotherapeutic drugs by means of MSNs decorated with GA (Figure 1). The selected drugs are doxorubicin (DOX) [21] and camptothecin (CPT) [23]. This therapeutic combination has attracted considerable interest due to their potent inhibition of enzymes TOP2 and TOP1, respectively, leading to synergistic chemotherapy [9,24,25]. Unfortunately, the modest solubility of CPT hampers its clinical use and its loading within the MSNs [26,27]. Although more soluble analogues such as topotecan or irinotecan are promising drugs, their lower cytotoxicity limits its use [28]. Therefore, it is proposed to prepare a pegylated CPT prodrug with enhanced solubility to be loaded into the MSNs instead of CPT [29]. Once the prodrug is inside of the MSNs, dihydrazide polyethylene glycol chains will be attached to the surface of an aldehyde-functionalized MSNs in order to block the voids by means of hydrazone bonds. The second dihydrazide group will be reacted with the ketone moiety present at the DOX structure. To endow the DDS with selectivity toward HepG2 cells, the MSN will be decorated with GA by means of imine bonds taking advantage of the presence of amino group on the tetrahydropyran ring attached to DOX. Hence, the DDS presents two different cleavable bonds. The imine bond located at the outer part of the system that will be cleaved at the slightly acidic pH (6.8) [30], and a hydrazone group which is cleaved at more acidic pH (4.5) typical of endosomes (Figure 1).

## 2. Materials and Methods

### 2.1. Materials and Measurements

Tetraethyl orthosilicate (TEOS), 3-aminopropyl triethoxisilane (APTES), cetyltrimethylammonium bromide (CTAB), toluenesulfonyl chloride, and 18-beta-Glycyrrhetinic acid (GA) were purchased from ACROS (Geel, Belgium); ammonium hydroxide solution (2,2-dimethoxyethan-1-amine; hydrazine hydrate, tetraethylene glycol, 4-hydroxybenzaldehyde, succinic anhydride, anhydrous pyridine, 4-(dimethylamino)pyridine (DMAP), dichloromethane (DCM), cyclohexane (Cy); ethyl acetate (AcOEt), 2,5,8,11-tetraoxatridecane-13-thiol, tetraethylene glycol monotosylate, hexane, EtOH, toluene, DMSO, MeOH, NH_4_NO_3,_ NH_3_, *N*,*N*′-dicyclohexylcarbodiimide (DCC), triphosgene, potassium carbonate, acetonitrile, doxorubicin hydrochloride, 2,2′-dipyridyldisulphide (>98%), 1,1′-thiocarbonyldi-2(1*H*)-pyridone (95%), 2-mercaptoethanol (98%), DAPI, and Anti-Rab7 antibody from Sigma Aldrich (St. Louis, MO, USA); goat anti-rabbit IgG H&L (Alexa fluor^®^ 555) was purchased from abcam (Cambridge, UK); Triton-x-100, formalin, MTT, DMEM, FBS, glutamine, penicillin, streptomycin, tripsyn, and PBS were purchased from Labclinics, Barcelona, Spain); camptothecin was purchased from Apollo (Stockport, Cheshire, UK). All the chemicals were used as received without further purification.

### 2.2. Instruments

Fluorescence Confocal Microscopy images were acquired using a multiphoton spectral Leica model SP8 lighting. Flow cytometry analysis was carried out on a flow cytometer NovoCyte^®^ (ACEA Biosciences, Inc., Santa Clara, CA, USA). Dynamic Light Scattering (DLS) size and ζ-potential determinations were obtained using a Malvern Zetasizer Nano Series ZEN 3600 (Malvern, UK). UV–vis absorption spectra were recorded using a Thermo Scientific 300 UV-vis spectrophotometer (Waltham, MA USA). Nuclear Magnetic Resonance spectra (^1^H-NMR and ^13^C-NMR) were recorded on a Varian 400-NMR spectrometer (Varian, Inc., Palo Alto, CA, USA) (^1^H-NMR at 400 MHz and ^13^C-NMR at 100.6 MHz). Coupling constants are reported in Hertz (Hz). Spectral splitting patterns are designed as follows: s (singlet), d (doublet), t (triplet), q (quartet), dd (doublet of doublets), ddd (doublet of doublet of doublets), m (complex multiplet), and brs (broad signal).

High Resolution Mass Spectrometry (HRMS) was performed on a VG AutoSpec (Micromass Instruments, Waters, Corp., Milford, MA, USA) Trisctor EBE of high-resolution spectrometer operating in FAB or EI mode and on Biotoff II (Bruker, Billerica, MA, USA) apparatus in ESI-TOF mode. The spectra were recorded at “Servicio de Espectroscopia de Masas, Universidad de Santiago de Compostela”.

### 2.3. Synthesis of 2-(Pyridyl-Disulfanyl)ethanol (**4**)

0.3 g (1.36 mmol) of 2,2′-pyridildisulphide was dissolved in DCM (5 mL) and 78 μL (64 mg, 0.82 mmol) of 2-mercaptoethanol dissolved in DCM (5 mL) was added to the mixture dropwise over 30 min. The mixture was stirred overnight. The result crude was purified through a silica gel column chromatographic with hexanes:AcOEt mixtures 8:2. The product **4** was collected and the solvent was removed by rotatory evaporation [31]. Yield: (**4**) 96.9 mg (ƞ = 38%).

**^1^H NMR** (400 MHz, CDCl_3_) *δ(ppm):* δ 8.49 (d, *J* = 4.7 Hz, 1H), 7.57 (t, *J* = 8.1 Hz, 1H), 7.40 (d, *J* = 8.0 Hz, 1H), 7.19–7.08 (m, 1H), 3.85–3.74 (m, 2H), 2.98–2.87 (m, 2H).

### 2.4. Synthesis of Camptothecin-(4-Pyridyldisulfanyl)ethyl Carbonate (***2***)

A solution of 167 mg (1.36 mmol) of DMAP, 100 mg (0.33 mmol) of CPT, and 55 mg (0.18 mmol) of triphosgene was dissolved in anhydrous DCM (30 mL). The solution was reacted at room temperature. After 15 min, 55 mg (0.18 mmol) of compound **4** was added to the solution, and the mixture was stirred vigorously at room temperature for 18 h under argon atmosphere. The reaction was quenched by washing the organic layer with brine and dried using anhydrous MgSO_4_. The result crude was purified through a silica gel column chromatographic with AcOEt. Compound **2** was collected, and the solvent was removed by rotatory evaporation to yield the pure product as a yellow solid. Yield: (**2**) 91 mg (ƞ = 98%).

**^1^H NMR** (400 MHz, CDCl_3_) *δ(ppm):* 8.54 (s, 1H), 8.50 (s, 1H), 8.37 (d, *J* = 8.6 Hz, 1H), 7.98 (d, *J* = 7.4 Hz, 2H), 7.89 (t, *J* = 7.7 Hz, 1H), 7.72 (q, *J* = 7.1, 6.7 Hz, 1H), 7.58 (s, 1H), 7.34 (s, 1H), 5.69 (d, *J* = 17.3 Hz, 1H), 5.39 (d, *J* = 17.4 Hz, 1H), 5.34 (s, 2H), 4.35 (t, *J* = 6.0 Hz, 2H), 3.18 (t, *J* = 6.0 Hz, 2H), 2.27 (m, 1H), 2.17 (m, 1H), 1.01 (t, *J* = 7.2 Hz, 3H).

### 2.5. Synthesis of (S)-4-Ethyl-3,14-Dioxo-3,4,12,14-Tetrahydro-1H Pyrano [3′,4′:6,7] Indolizino [1,2-b]Quinolin-4-yl (2,5,8,11-Tetraoxa-14,15-Dithiaheptadecan-17-yl) Carbonate (***1***)

50 mg (0.08 mmol) of camptothecin-(4-pyridyldisulfanyl)ethyl carbonate (**2**) was dissolved in DCM. Moreover, 20 mg (0.08 mmol) of 2,5,8,11-tetraoxatridecane-13-thiol was dissolved in DCM (5 mL) and added to the solution dropwise over 30 min. The mixture was stirred for 6 h at room temperature. The result crude was purified through a silica gel column chromatographic with AcOEt. The product **1** was collected and the solvent was removed by rotatory evaporation. Yield: (**1**) 43 mg (ƞ = 75%).

**^1^H NMR** (400 MHz, CDCl_3_) *δ (ppm)*: 8.41 (s, 1H), 8.25 (d, *J* = 8.5 Hz, 1H), 7.95 (d, *J* = 8.1 Hz, 1H), 7.85 (ddd, *J* = 8.4, 6.8, 1.4 Hz, 1H), 7.68 (t, *J* = 7.5 Hz, 1H), 7.36 (s, 1H), 5.70 (d, *J* = 17.2 Hz, 1H), 5.39 (d, *J* = 17.3 Hz, 1H), 5.31 (s, 2H), 4.37 (d, *J* = 7.2 Hz, 1H), 3.63 (s, 5H), 3.36 (s, 3H), 2.93 (td, *J* = 6.9, 1.7 Hz, 2H), 2.84 (t, *J* = 6.5 Hz, 2H), 2.29 (dd, *J* = 14.2, 7.3 Hz, 1H), 2.15 (dd, *J* = 14.2, 7.3 Hz, 1H), 1.00 (t, *J* = 7.5 Hz, 3H).

**^13^C NMR** (100 MHz, CDCl_3_) *δ (ppm)*: 167.4, 157.4, 153.6, 152.3, 148.8, 146.4, 145.7, 131.5, 130.9, 129.9, 128.6, 120.5, 96.4, 78.1, 77.4, 72.1, 70.74, 70.6, 70.5, 69.5, 67.2, 66.8, 59.2, 50.2, 38.7, 36.5, 32.0, 7.8.

**ESI-MS *m*/*z***: calcd. 674.20 found 675.20 (M−H).

### 2.6. Synthesis of 4-(2-(2-(2-(2-Hydroxyethoxy) Ethoxy) Ethoxy) Ethoxy) Benzaldehyde (***5***)

0.73 g (6 mmol) of 4-hydroxybenzaldehyde, 2.10 g (6 mmol) of tetraethylene glycol monotosylate, and 2.50 g (18.1 mmol) of K_2_CO_3_ were dissolved in dry MeCN (50 mL). The solution was heated at reflux under a nitrogen atmosphere for 3 days. After the addition of CH_2_Cl_2_ (50 mL), the mixture was filtered and the precipitate was washed with CH_2_Cl_2_ (2 × 50 mL). The organic filtrates were combined and the solvents were evaporated to afford the crude as a pale orange oil. The product **5** was used in the next step without further purification. Yield: (**5**) 1.80 g (ƞ = 99%).

**^1^H NMR** (400 MHz, CDCl_3_) *δ (ppm)*: 2.60 (t, *J* = 5.9 Hz, 1H), 3.77–3.56 (m, 12H), 3.92–3.87 (m, 2H), 4.25–4.20 (m, 2H), 7.05–6.99 (m, *J* = 8,4 Hz, 2H), 7.86–7.80 (m, *J* = 8.4 Hz, 2H).

### 2.7. Synthesis of 10-((3-Carboxypropanoyl)oxy)-2,4a,6a,6b,9,9,12a-Heptamethyl-13-oxo-1,2,3,4,4a,5,6,6a,6b,7,8,8a,9,10,11,12,12a,12b,13,14b-Icosahydropicene-2-Carboxylic Acid (***6***)

4.7 g (10.0 mmol) of 18-β-glycyrrhetinic acid, 2.2 g (22.0 mmol) of succinic anhydride, and anhydrous pyridine (80 mL) was stirred with heating at 80 °C for 48 h. Then, the solvent was removed *in vacuo* and the residue was dissolved in DCM (80 mL), washed with diluted HCl (1 mol/L, 20 mL × 3), and extracted with CH_2_Cl_2_ (30 mL × 3). The organic layers were combined and dried with MgSO_4_. The solvent was removed by rotatory evaporation, and the residual solid was purified by silica column chromatograph (hexanes:AcOEt = 1:1) to afford product **4** [32]. Yield: (**4**) 3.5 g (ƞ = 61%).

**^1^H NMR** (400 MHz, CDCl_3_) *δ (ppm):* 0.83 (s, 3H), 0.90 (s, 6H), 1.14 (s, 3H), 1.18 (s, 3H), 1.24 (s, 3H), 1.38 (s, 3H), 2.38 (s, 1H), 2.66–2.74 (d, 4H), 2.80 (d, 1H), 4.55–4.59 (dd, 1H).

### 2.8. Synthesis of 10-((1-(4-Formylphenoxy)-13-oxo-3,6,9,12-Tetraoxahexadecan-16-oyl)oxy)-2,4a,6a,6b,9,9,12a-Heptamethyl-13-oxo-1,2,3,4,4a,5,6,6a,6b,7,8,8a,9,10,11,12,12a,12b,13,14b-Icosahydropicene-2-Carboxylic Acid (***3***)

50 mg (0.08 mmol) of acid **6** and 23 mg (0.07 mmol) of aldehyde **5** was dissolved in 4 mL of anhydrous CH_2_Cl_2_. The reaction mixture was agitated for 10 min at 0 °C. Then, 13 mg (0.1 mmol) of DMAP and 18 mg (0.08 mmol) of DCC were dissolved in 5 mL of anhydrous CH_2_Cl_2_. This solution was added to the latter and keep overnight at room temperature under nitrogen atmosphere. Finally, the organic layer was washed with water (2 × 50 mL), dried with MgSO_4_, filtered, and evaporated *in vacuo* to afford compound **3**. Yield: (**3**) 47 mg (ƞ = 64%).

**^1^H NMR** (400 MHz, CDCl_3_) *δ (ppm)*: 9.87 (s, 1H), 7.86–7.77 (m, *J =* 8.4 Hz, 2H), 7.00 (m, *J* = 8.4 Hz, 2H), 5.67 (s, 1H), 4.52 (dd, *J* = 11.6, 4.8 Hz, 1H), 4.26–4.18 (m, 4H), 4.14 (d, *J* = 9.6 Hz, 2H), 3.91–3.85 (m, 2H), 3.76–3.56 (m, 8H), 3.51–3.39 (m, 4H), 2.83–2.74 (m, 1H), 2.68–2.59 (m, 2H), 2.38 (s, 1H), 1.38 (s, 3H), 1.24 (s, 3H), 1.18 (s, 3H), 1.14 (s, 3H), 0.90 (s, 6H), 0.83 (s, 3H).

**^13^C NMR** (100 MHz, CDCl_3_) *δ (ppm)*: 200.3, 190.9, 172.2, 170.2, 163.9, 157.5, 132.1, 130.1, 128.4, 114.9, 81.2, 71.4, 70.3, 69.3, 67.8, 63.9, 61.7, 55.1, 49.6, 46.8, 45.5, 43.3, 39.6, 38.2, 36.9, 33.8, 31.9, 29.8, 27.6, 27.0, 24.7, 23.5, 18.8, 17.7, 16.2.

**ESI-MS *m*/*z***: calcd. 850.49 found 851.49 (M−H).

### 2.9. Synthesis of MSN-(NH_2_) CTAB

An ethanolic solution of 1.6 mL of TEOS (0.2 M) was added dropwise to a 100 mL of CTAB solution in NH_3(aq)_ (0.5 M) at 60 °C. Then, this mixture was stirred for 5 h. Afterwards, 1.6 mL of APTES dissolved in EtOH (12% *v/v*), and 1.6 mL of TEOS in EtOH (1 M) were added dropwise. Subsequently, the resulting mixture was stirred at the same temperature for 1 h. Finally, the particles were aged for 24 h. The as-synthetized nanoparticles were collected via centrifuging, washing, and dispersing with deionized H_2_O [5].

### 2.10. Synthesis of MSN-(NH_2_)_i_(CHO)_o_

MSN-(NH_2_) CTAB (0.2 g) were mixed with 0.1 g of 2-isothiocyanate-1,1-dimethoxyethane (prepared as described in ref. [5]) (4 eq 0.1 g, 6.8 × 10^−4^ mol) in 50 mL of dry toluene. After 24 h, the resulting solid was washed twice with toluene and EtOH. Then, the tensioactive was removed by treatment of MSN-(NH_2_)_i_(Acet)_o_ with NH_4_NO_3_ (0.5 g) in EtOH (40 mL) at 25 °C. After 24 h, the MSN were washed with EtOH. Finally, the acetal group was removed by treatment of the nanoparticles with 0.1 M HCl (30 mL) for 6 h [5].

### 2.11. Synthesis of CPT-PEG@MSN-DOX

CPT-PEG (18 mg, 2.66 × 10^−5^ mol) was dissolved in CHCl_3_ (35 mL) with sonication at 60 °C. Then, CPT-PEG was added to a suspension of MSN-(NH_2_)_i_(CHO)_o_ (30 mg) in MeOH (25 mL) with stirring (700 rpm, 23 °C). Afterwards (24 h), a solution of 3,6,9,12,15-pentaoxaheptadecanedihydrazide (0.080 g, 2.36 × 10^−4^ mol) in MeOH (20 mL) was added. After an additional 24 h, the resulting particles were centrifuged and washed with the mixture CHCl_3_/MeOH (4:1) twice. Then, the supernatant was collected in order to quantify the loading of the drug by UV–vis spectroscopy (354 nm). Then, the as-synthesized nanoparticles were dispersed in 20 mL of a methanolic solution of DOX (21 mg, 3.6 × 10^−5^ mol). Finally, after 48 h, the particles were washed with MeOH (3 times) and H_2_O (3 times) until no red supernatant was obtained and lyophilized. DOX and CPT loading content (%LC = [Entrapped Drug/nanoparticles weight] × 100) was quantified by UV–vis spectroscopy (354 nm and 490 nm, respectively) from the supernatant (the amount of prodrug released to the supernatant during the pore capping was subtracted from the initial value of loaded drug (**CPT-PEG@MSN-(NH_2_)_i_(CHO)_o_**)).

### 2.12. Conjugates CPT-PEG@MSN-DOX-OH and CPT-PEG@MSN-DOX-GA

5 mg of **CPT-PEG@MSN-DOX** was placed in a round-bottomed flask with 10 mL of basic water (pH 8.5). Then, an excess of aldehyde **5** or **3** was added to the solution. The mixture was stirred at room temperature for 24 h. Solid samples were collected by centrifugation at 13,000 rpm for 13 min, washing with basic water (pH 8.5), twice. Solvent was eliminated and MSNs were stored dry yielding **CPT-PEG@MSN-DOX-OH** and **CPT-PEG@MSN-DOX-GA**.

### 2.13. Release Experiments of CPT-PEG@MSN-DOX-GA

The release of the drugs from the particles were assessed at different pH values: 7.4 (PBS buffer), 6.5 (phosphate buffer, 0.2 M NaH_2_PO_4_/0.2 M Na_2_HPO_4_), 5.5, 4.5, and 4 (acetate buffer, 0.1 M NaAcO/0.1 M AcOH). For each determination, 10 mg of the particles were dispersed by sonication in 1.5 mL of the buffer solution during 2 min at 37 °C with stirring (100 rpm). Then, samples of the solution were taken out and centrifuged for 13 min at 12,000 rpm. The residues were dispersed in fresh buffer solution (1.5 mL). The quantity of the drug released was measured by UV–vis absorption spectroscopy at 490 nm and 345 nm for DOX and CPT, respectively.

### 2.14. In Vitro Cytotoxicity

In vitro cytotoxicity of the DDSs was assessed using the MTT assay according to literature procedures. The experiments were performed in 96-well plates (0.1 mL/well). A total of 10,000 cells/well were seeded using complete DMEM media. Briefly, after 24 h, cells were incubated with the corresponding nanoparticles for 24 to 72 h at 37 °C and 5% CO_2_. The cells were washed with PBS buffer and then a solution of MTT reagent (3-(4,5-dimethylthiazol-2-yl)-2,5-diphenyl tetrazolium bromide) in (0.5 mg/mL) was added. Subsequently, cells were incubated for 3 h at 37 °C (5% CO_2_). Afterwards, the solvent was removed. The formazan crystals formed were dissolved in 0.1 mL of DMSO and the concentration of the dye was determined (560 nm). The absorbances were normalized to negative controls to determine cellular viability.

### 2.15. Confocal Microscopy for Cellular Internalization

Uptake experiments were performed on gelatin-treated glass coverslips. A total of 200,000 HepG2 cells/well were seeded using complete DMEM media (10% FBS, 1% glutamine, and 1% penicillin/streptomycin). After 24 h, cells were incubated with 100 µg·mL^−1^ of **CPT-PEG@MSN-DOX**, **CPT-PEG@MSN-DOX-OH**, and **CPT-PEG@MSN-DOX-GA** for 1 h, 4 h, and 10 h at 37 °C and 5% CO_2_. For immunofluorescence, cells were fixed with 10% formalin in PBS for 10 min at room temperature. After 3 washing steps with PBS, cells were permeabilized using 0.1% Triton-X-100 in PBS for 10 min at room temperature. Then, a blocking step was performed using 5% milk powder in PBS for 1 h at room temperature. Subsequently, cells were incubated with mouse monoclonal anti-Rab7 primary antibody diluted in the blocking solution overnight at 4 °C followed by three PBS washing steps. The secondary antibody, goat anti-mouse Alexa fluor 555, was diluted in the blocking solution and incubated for 1 h at room temperature. Afterwards, nuclei were stained using DAPI at 1:1000 for 10 min at room temperature. Finally, cells were observed in a laser-scanning confocal spectral microscopy (Leica model SP8 lighting) with Argon and HeNe lasers attached to a Leica DMI6000 inverted microscope. Images were acquired using an APO 40× objective lens. Numerical aperture: 1.4, 405, 488, and 532 nm laser lines. Emission was detected in the range of 415–460 nm, 500–525 nm, and 550–600 nm at a 512 × 512 format and zoom 1.

### 2.16. Flow Cytometry Analysis

HeLa and HepG2 cells were seeded at a density of 20,000 cells/well in 96-well plates with complete DMEM media (10% FBS, 1% glutamine, and 1% penicillin/streptomycin) and incubated at 37 °C, 5% CO_2_ for 24 h. Then, 100 µg·mL^−1^ of the corresponding nanoparticles (**CPT-PEG@MSN-DOX**, **CPT-PEG@MSN-DOX-OH**, and **CPT-PEG@MSN-DOX-GA**) were added, and after a 1-h, 4-h, 10-h, and 24-h incubation period, cells were prepared for analysis. To do so, cells were washed with PBS and harvested with 40 μL of trypsin per well. After 5 min, 120 μL of complete DMEM were added and cells were fixed with 100 μL of paraformaldehyde 2%. Then, DOX internalization was measured (488 nm).

## 3. Results and Discussion

### 3.1. Synthesis of CPT Prodrug and Cleavage

**Scheme 1:**
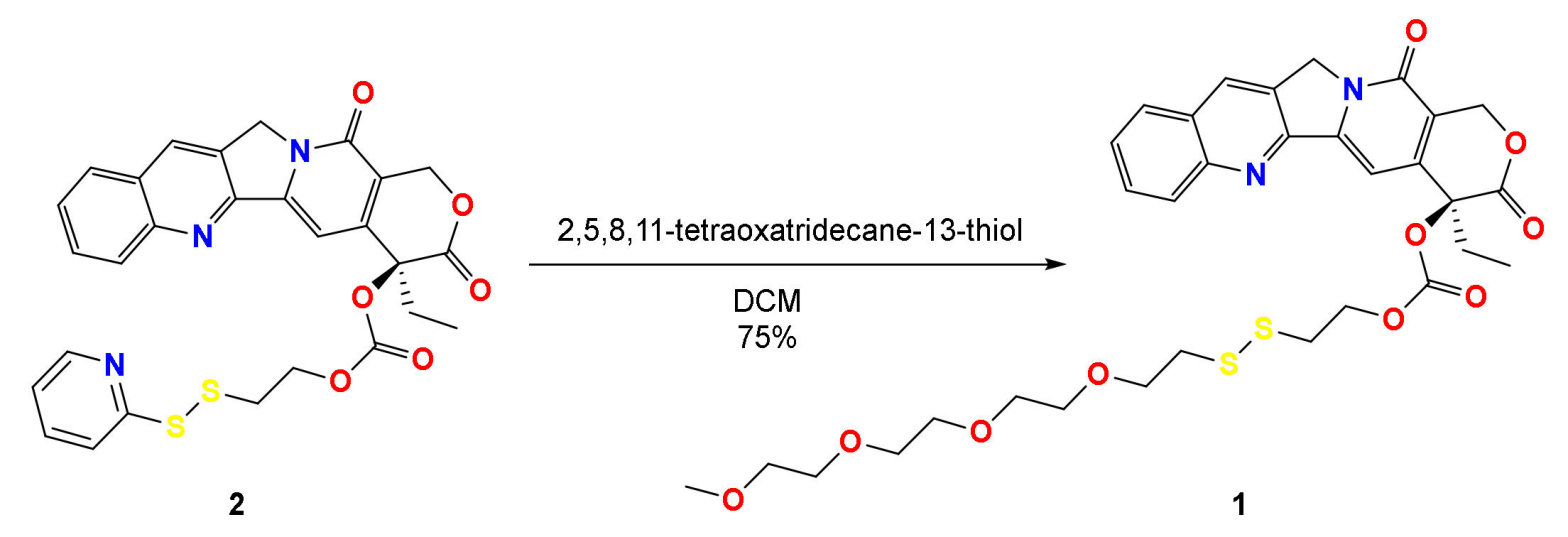
Synthesis of camptothecin (CPT)-PEG (**1**).

As mentioned, the main limitation of CPT for its translation into clinics is its low solubility. This drawback hampers not only the treatments but also the loading and release of the drug from DDSs. To circumvent these drawbacks, in this work, the use of a prodrug is proposed. Such molecule should encompass two features: enhanced amphiphilicity compared to CPT and similar cytotoxicity. A general methodology to impart solubility to hydrophobic drugs is pegylation. However, the presence of a PEG (Polyethylene glycol) chain could be detrimental for the biological activity of CPT. Thus, it is proposed that the PEG chain could be tethered to the drug using a cleavable glutathione-sensitive linker [27,33]. A search into the literature revealed that a cleavable traceless linker can be easily installed into the structure of CPT enabling the pegylation of the drug [34]. Thus, the synthesis of prodrug **1** is proposed (Scheme 1). The preparation of **1** entails the reaction of commercially available 2,5,8,11-tetraoxatridecane-13-thiol with (pyridin-2-yldisulfanyl)alkyl carbonate derivative **2** [31] to furnish prodrug CPT-PEG **1** in 75% yield after silica column gel chromatography purification (Figures S1 and S2).

The formation of CPT from the prodrug by the cleavage of the disulphide bond was studied in vitro using ^1^H-NMR. It is reported that the concentration of glutathione (GSH) inside cancerous cells is in the range of 2–8 mM, while in plasma it is only 1–2 μM. Hence, in order to simulate the intracellular conditions, the prodrug was treated with GSH 10 mM, in PBS buffer pH = 7.4. From inspection of the spectra (Figure S3) a shift of the triplet ascribed to the CH_3_CH_2_ group of CPT from 1.01 to 1.05 ppm (red dot) can be observed. Further evidence of the cleavage was given by the appearance of a triplet at 2.70 ppm (green dot), which corresponds to the protons of the CH_2_–SH group of the PEG chain. Under these experimental conditions, it was found that in 1 h all the prodrug was transformed into free CPT [27].

### 3.2. Construction of the System CPT-PEG@MSN-DOX

Once in the hand of prodrug **1**, the DDS was built using regioselectively functionalized amino-aldehyde MSNs. The synthesis of these nanoparticles has been previously reported by our group [5,19] (Tables S1 and S2, Figure S4). Then, CPT-PEG (**1**) was loaded into the pores of the MSNs using a mixture of CHCl_3_/MeOH (4:1) as solvent. A maximum loading of 9.33 × 10^−8^ mol CPT-PEG·mg^−1^ MSN was achieved, which corresponds to a 6.3 ± 0.3% (mg de CPT-PEG/mg de MSN). It must be highlighted that this yield represents a 2.3 ± 0.4% of increase of the loading in respect to that obtained when CPT was loaded under the same conditions. Hence, in principle, after a reductive cleavage of the PEG chain, this increment corresponds to a 30% of increase of CPT available.

Subsequently, an excess of 3,6,9,12,15-pentaoxaheptadecanedihydrazide [5] (Scheme S1) was added to the suspension to render the corresponding mono hydrazone. Then, the unreacted reagent was eliminated by centrifugation to avoid any unwanted reaction in the next step. Finally, the nanoparticles were treated with DOX in EtOH as solvent. The drug reacted with the available hydrazide group of the PEG chain yielding a second hydrazone bond Approximately, 7.06 × 10^−7^ mol DOX·mg^−1^ MSN were incorporated to the MSNs, which corresponds to a loading of about 25% (mg de DOX/mg de MSN).

### 3.3. Construction of the DDS (CPT-PEG@MSN-DOX-GA)

To improve the tumor targetability and efficiency of the system, **CPT-PEG@MSN-DOX** particles were decorated with GA. The strategy of derivatization takes advantage of the presence of an amine group at the pyrane ring of DOX. The aim is to attach GA to the nanoparticle through an imine bond, which will be easily hydrolyzed under mild acidic conditions. In this way, both GA and DOX molecules will act as stoppers removable by a pH stimulus.

**Scheme 2:**
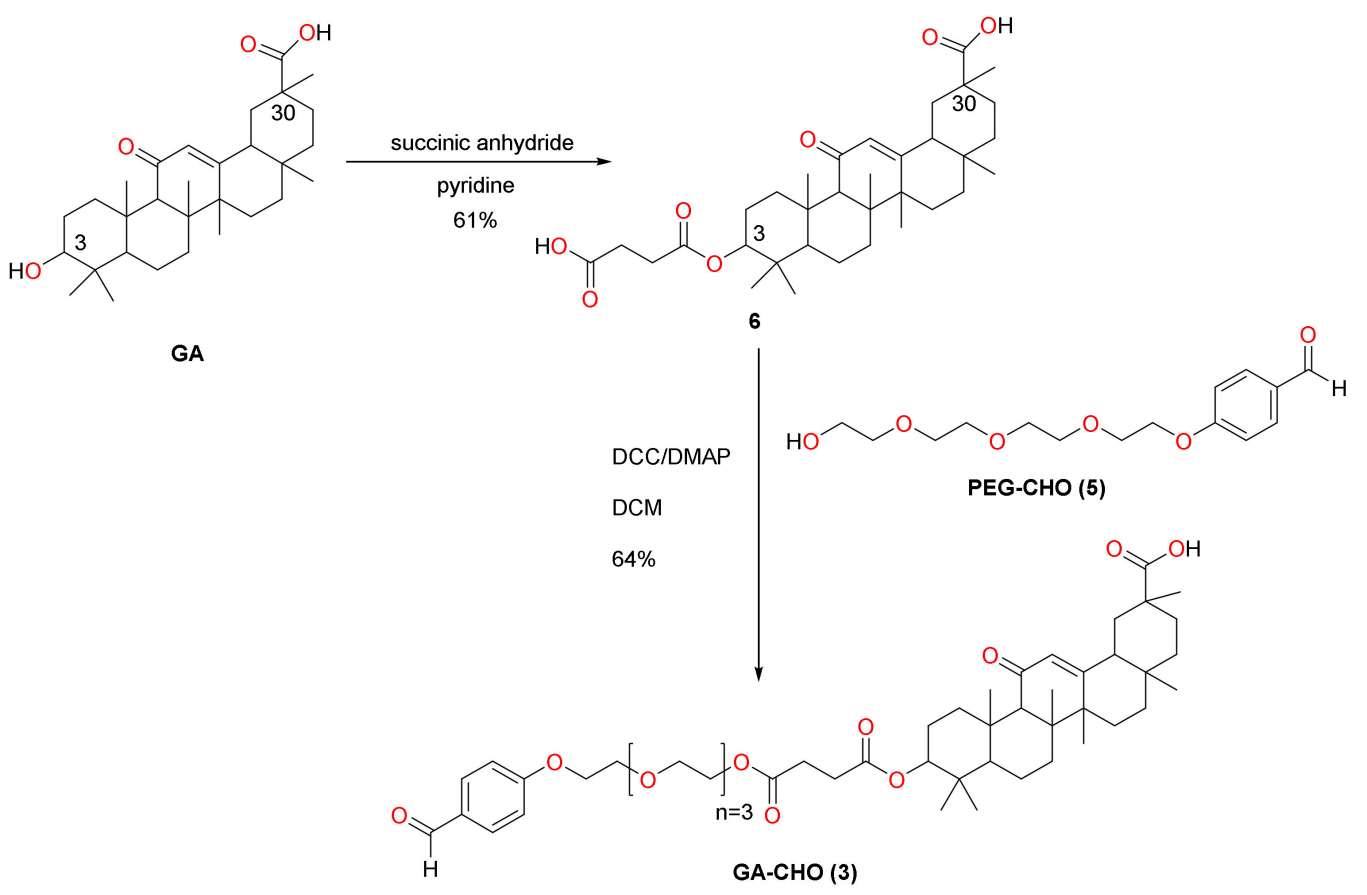
Synthetic pathway for the preparation of glycyrrhetinic acid (**GA)-CHO** (**3**).

First, GA was derivatized with a benzaldehyde moiety linked by a PEG chain (Scheme 2, Figure S5). Although GA bears a carboxylic acid in its structure suitable for conjugation, it is reported that this group is essential for an effective targeting [22,35]. Hence, a carboxylic moiety was attached at C3 of GA by esterification of the alcohol with succinic anhydride (Figures S6 and S7). In the following step, the newly installed carboxylic acid was reacted selectively with a bifunctional PEG bearing a benzaldehyde moiety and an alcohol group (**PEG-CHO**, Scheme 2) by means of Steglich reaction to furnish aldehyde **GA-CHO** (**3**) in 64% yield [36].

**Scheme 3:**
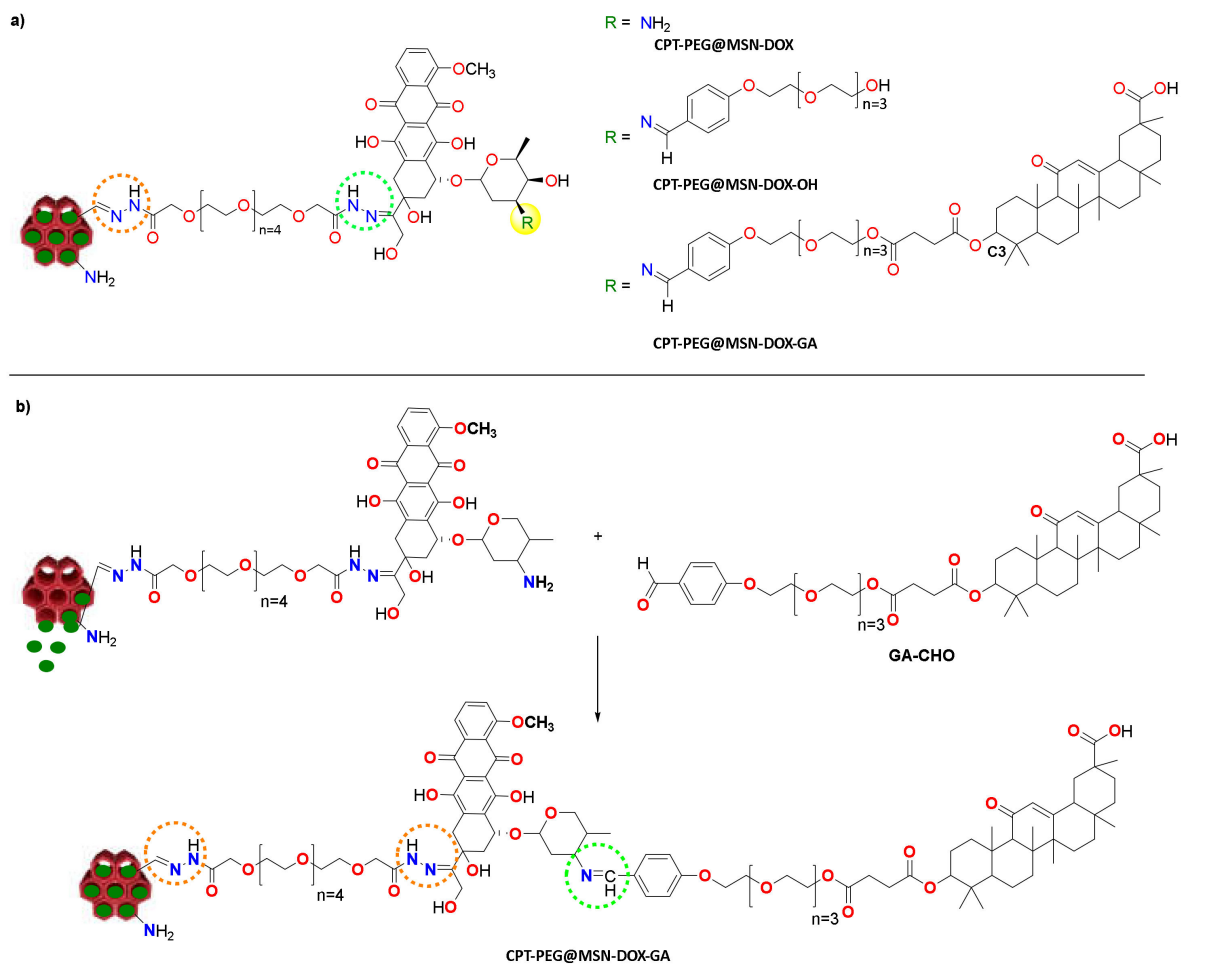
(**a**) Schematic representation of **CPT-PEG@MSN-DOX**, **CPT-PEG@MSN-DOX-OH**, and **CPT-PEG@MSN-DOX-GA** and (**b**) synthesis of **CPT-PEG@MSN-DOX-GA**.

Finally, **3** was attached to the nanoparticle by imine formation by reaction with benzaldehyde **GA-CHO**, the amino group of DOX (Scheme 3). The estimated amount of GA conjugated is about 0.60 mg GA/mg MSN.

**Figure 2:**
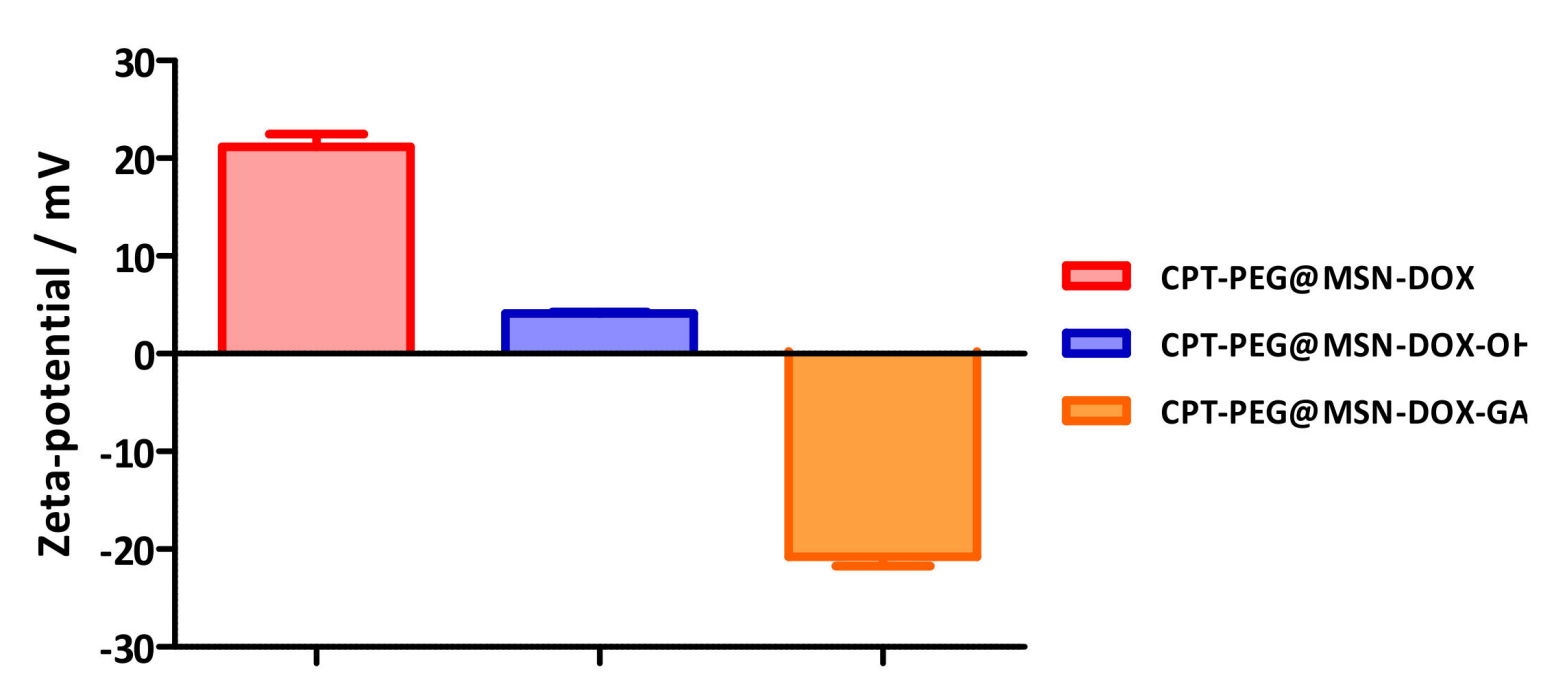
Zeta potential of **CPT-PEG@MSN-DOX**, **CPT-PEG@MSN-DOX-OH**, and **CPT-PEG@MSN-DOX-GA**. Data represented as mean ± SD (*n* = 3). Data measurements at pH = 5.5.

To verify the correct introduction of the GA ligand onto the DDS, the zeta potential of the particles was determined (Figure 2). Thus, system **CPT-PEG@MSN-DOX** showed a zeta potential of +21 mV. This value is consistent with the presence of the protonated amino group bonded to the pyrane moiety of DOX. In contrast, for **CPT-PEG@MSN-DOX-GA**, the potential was reversed, displaying a value of −20 mV, which is in accordance to reported surface charges in the literature for similar systems. The change of the potential stems from the presence of a carboxylic acid functionality in the GA [17]. As reference, MSNs functionalized with a PEG chain, **CPT-PEG@MSN-DOX-OH**, were prepared (Scheme 3a). As expected, the recorded zeta potential was close to neutrality, +4 mV [37].

The dependence of the zeta potential with the pH was monitored in a range of pHs from 8.0 to 5.0 (Figure S8). Under alkaline conditions, the potential was stabilized in an interval of −20 to −15 mV. Then, the potential abruptly raised at pH = 7.0 from −15 to +10 mV, at pH = 6.5. At more acidic conditions, the potential gradually reached higher values being the zeta potential at pH = 5.5 of +21 mV. This behavior is in agreement with the hydrolysis of the imine bond at mild acidic conditions rendering a protonated amino group on DOX molecule.

### 3.4. Controlled Drug Release

**Figure 3:**
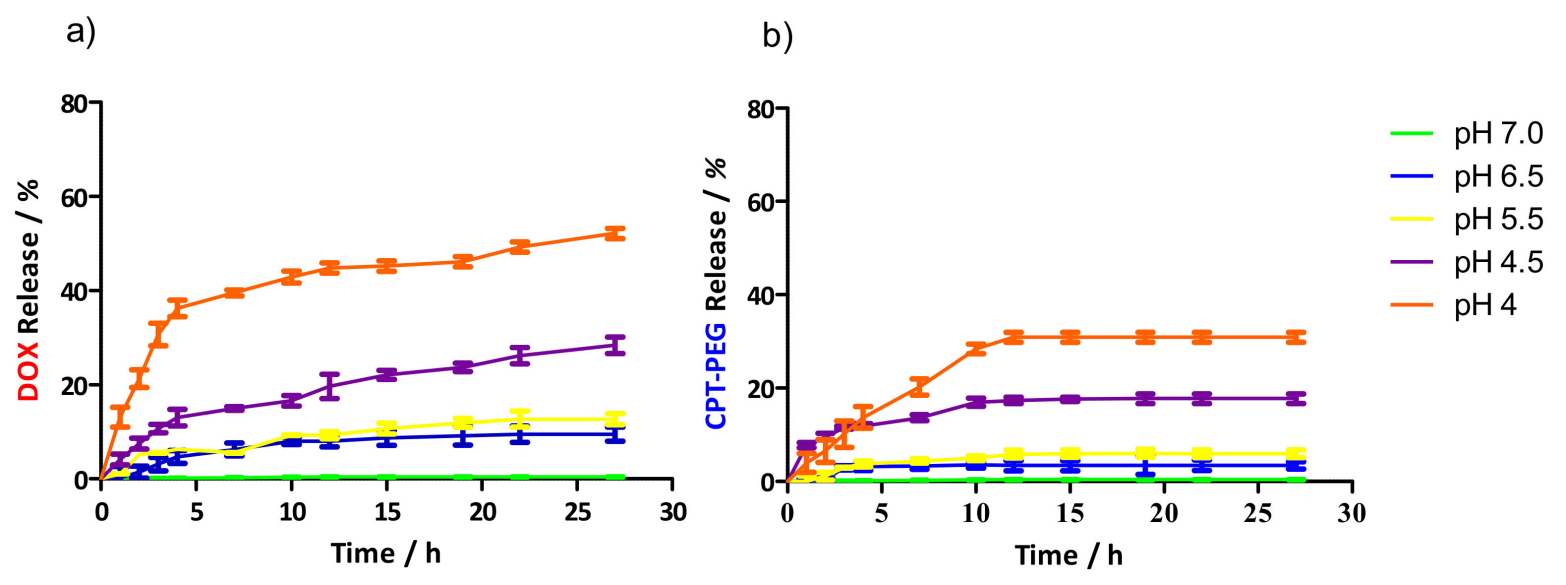
(**a**) DOX and (**b**) CPT-PEG release profile of **CPT-PEG@MSN-DOX-GA** at different pH values under stirring at 100 rpm and *t* = 37 °C. Data represented as mean ± SD (*n* = 3).

The release profiles of CPT-PEG (**1**) and DOX from **CPT-PEG@MSN-DOX-GA** were studied by UV–vis absorption spectroscopy at pH values of 4.0, 4.5, 5.5, 6.5, and 7.4 (Figure 3). At neutral conditions (pH = 7.4), **CPT-PEG@MSN-DOX-GA** showed a negligible release of CPT-PEG or DOX, which proved the stability of the system under physiological conditions. In contrast, as expected, a gradual release of both drugs was observed under acidic conditions, which agrees with the scission of the labile hydrazine and imine bonds. At pH = 5.5, which is the approximate value of pH found in the late endosomes, DOX and CPT-PEG releases were 25% and 15% of the drug within 20 h, respectively. Although, a total release of the drugs was not observed within the range of pH studied, the exposure of the nanoparticles to a highly acidic medium (pH = 1.0) liberates almost the total amount of the cargo. It is hypothesized that the drugs are partially absorbed onto the silica nanoparticles. This observation has been noted in similar systems [38,39].

While in the cellular endosome the imine and the hydrazine bonds will be easily cleaved, their differential stability will play an important role in the internalization of the nanoparticles. According to the design of the DDS, in principle, the nanoparticles can be internalized by receptor-mediated endocytosis. However, an alternative mechanism arises from the fact that GA is tethered to the DDS through an imine bond, which is labile al pH found on the extracellular matrix of tumors (pH = 6.8). The hydrolysis of the imine would lead to a cationic amine which would contribute to the internalization process [40]. It is worth noting that these two mechanisms would not be possible in non-tumoral tissue. In any case, due to the π-conjugation of the aromatic imine, it is likely that the bond would remain stable. Hence, the MSN uptake is expected to be mainly driven by the ligand–receptor interaction.

### 3.5. Selectivity in the Cellular Uptake

**Figure 4:**
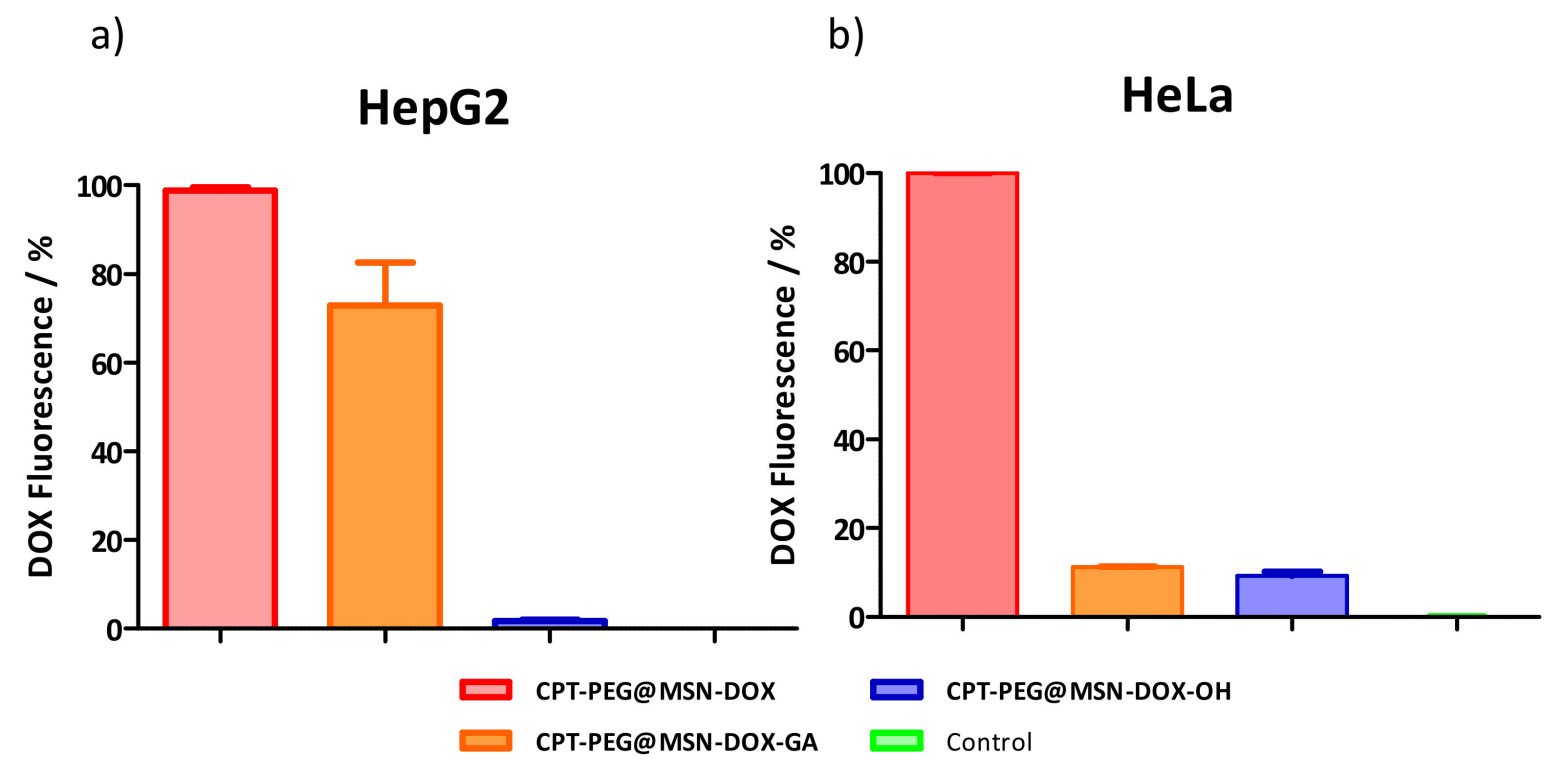
Flow cytometry analysis of HepG2 cells (**a**) and HeLa cells (**b**) incubated with **CPT-PEG@MSN-DOX**, **CPT-PEG@MSN-DOX-OH**, and **CPT-PEG@MSN-DOX-GA** for 4 h at 100 μg·mL^−1^. Untreated cells were used as control. Data represented as mean ± SD (*n* = 3).

Flow cytometry analysis was carried out in order to compare the uptakes of **CPT-PEG@MSN-DOX** and **CPT-PEG@MSN-DOX-GA** in HepG2 cells (GA-receptor positive cells). **CPT-PEG@MSN-DOX-OH** was used as control (Figure 4a). In addition, to assess the selectivity of the internalization, the same experiment was performed with HeLa cells as representative cell line, which does not contain GA receptors in its plasma membrane (Figure 4b). As expected, decorated nanoparticles with GA showed higher affinity for HepG2 than HeLa cells. More specifically, HepG2 cells displayed an enhancement of 73% of DOX fluorescence, while in HeLa cells, only an 11% was detected. Selectivity attained by the **CPT-PEG@MSN-DOX-GA** system to discriminate between cancer liver cells HepG2 and HeLa was about 9 to 1. On the other hand, **CPT-PEG@MSN-DOX** presents the highest uptake in both cell lines, with nearly 100% of DOX fluorescence, independently on the presence of GA receptors. It is hypothesized, that the high zeta potential value of such particles (+20 mV) is responsible for the disruption of the plasmatic membrane, enhancing the uptake in both cell lines. Finally, the internalization of **CPT-PEG@MSN-DOX-OH** was modest either in HepG2, 2% of DOX fluorescence, or HeLa cells, 9% of DOX fluorescence. Since this type of MSNs does not present any target ligand, a low internalization was expected for HepG2 cells [41]. Furthermore, its charge is not positive enough (+4 mV) to disrupt the membrane of HeLa cells.

From the results obtained, it can be stated that these MSNs follow different uptake pathways. It is hypothesized that **CPT-PEG@MSN-DOX** might undergo an endocytosis mediated by the electrostatic interaction, given the cationic character of the system [40]. As for nanoparticles, **CPT-PEG@MSN-DOX-GA** probably followed a receptor-mediated endocytosis pathway that would be consistent with the presence of the GA ligand.

**Figure 5:**
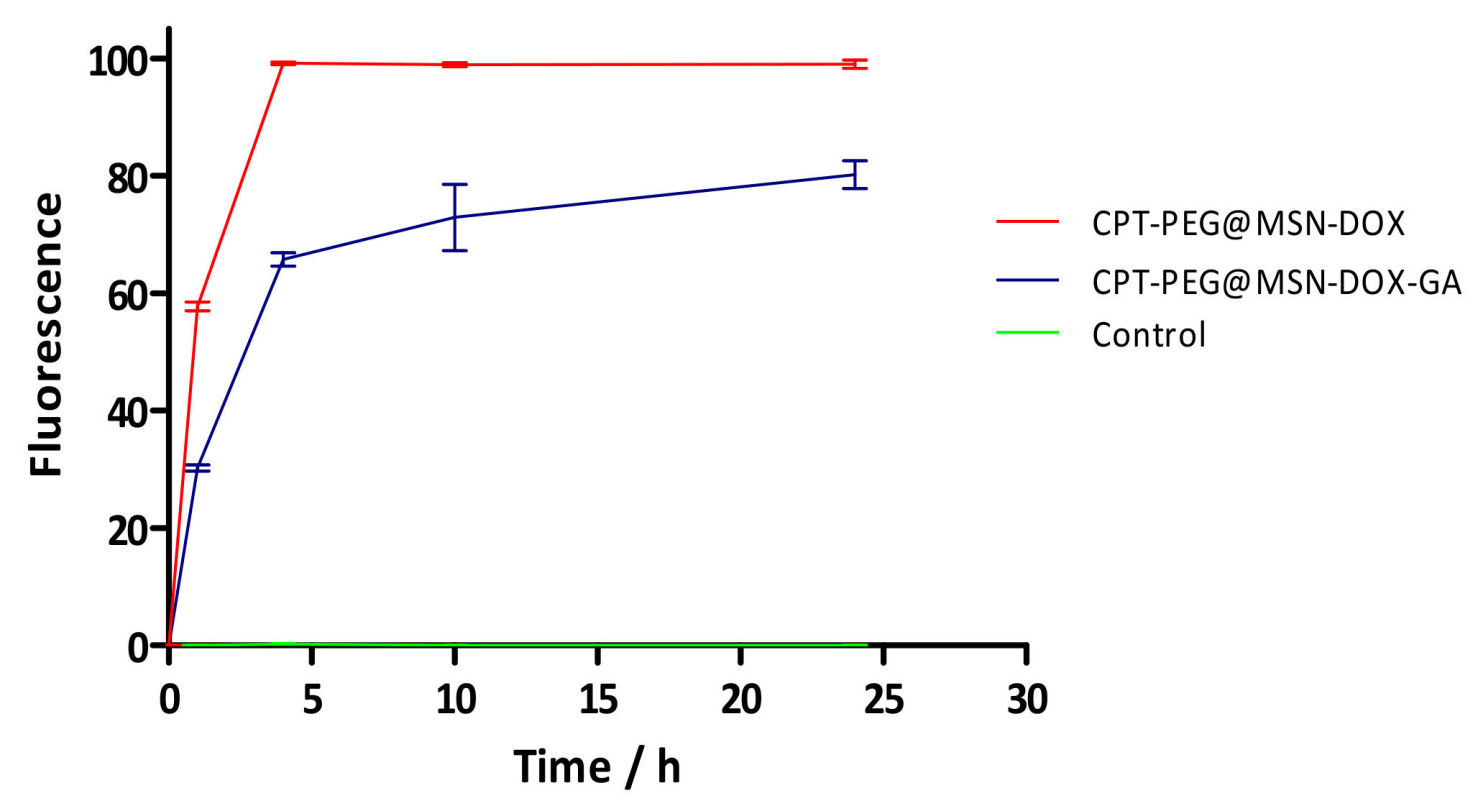
Kinetics of **CPT-PEG@MSN-DOX** and **CPT-PEG@MSN-DOX-GA** by Flow Cytometry analysis of HepG2 cells at 1, 4, 10, and 24 h at 100 μg·mL^−1^. Data represented as mean ± SD (*n* = 3).

In order to gain insight into the uptake of the systems, the internalization kinetics of **CPT-PEG@MSN-DOX** and **CPT-PEG@MSN-DOX-GA** were studied with HepG2 cells in a span of 24 h (Figure 5) [20]. To do so, the increase of the fluorescence of DOX was monitored by flow cytometry. As it can be observed in Figure 5, the untargeted system (**CPT-PEG@MSN-DOX**) quickly reaches a plateau after 4 h of incubation (100% of internalization). Whereas for the GA containing system (**CPT-PEG@MSN-DOX-GA**), the fluorescence gradually increases reaching the maximum uptake in 10 h, about 80%. It is worth noting that even at higher exposure times (24 h), the internalization did not increase, suggesting that the GA receptors were completely saturated, thus preventing endocytosis. Alternatively, this saturation can be induced by addition of an excess amount of **GA-CHO** (**3**) [3,17,21].

**Figure 6:**
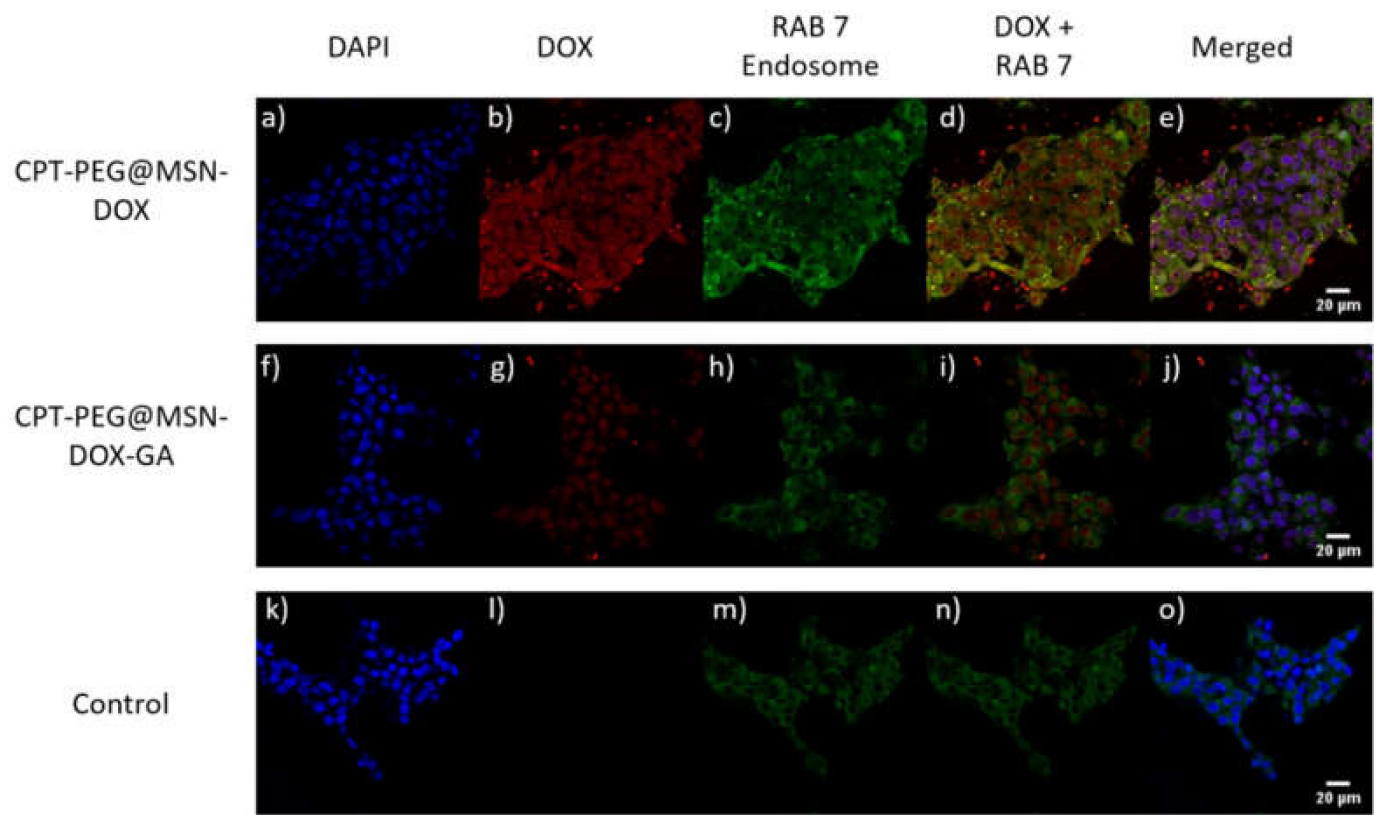
Confocal laser scanning microscopy (CLSM) images of the uptake for 1 h of **CPT-PEG@MSN-DOX** and **CPT-PEG@MSN-DOX-GA** (100 μg·mL^−1^) at 37 °C for 1 h at 100 μg·mL^−1^. Scale bar 20 μm. Blue: DAPI, red: DOX, green: RAB7.

The internalization of MSNs in HepG2 cells was further studied by confocal laser scanning microscopy (CLSM), labeling the endosomes of the cells with antibody against RAB7, a protein present in late endosomes, for two different periods of time: 1 h (Figure 6) and 10 h (Figure S9).

Inspecting Figure 6b,g, it can be determined that cells treated with untargeted MSNs displayed a higher intensity of DOX fluorescence in comparison with those treated with GA decorated MSNs. These results are in agreement with the kinetic study (Figure 5). Similar results were obtained after 10 h with the same systems (Figure S9b,g).

Surprisingly, DOX distribution in the cell organelles displayed different patterns when **CPT-PEG@MSN-DOX** or **CPT-PEG@MSN-DOX-GA** were administered to cells. In the first case, released DOX can be found either in the endosomes or the nucleus of the cell (Figure 6d) (yellow fluorescence due to their colocalization). In contrast, DOX fluorescence from **CPT-PEG@MSN-DOX-GA** was detected mainly within the nucleus of the cell (Figure 6i). These results were rationalized in terms of differences in the uptake and the endosomal escape capabilities of the MSNs.

It is hypothesized that the colocalization of endosomes and DOX released from **CPT-PEG@MSN-DOX** (Figure 6d) is accounted for the high amount of nanoparticles taken up by the cells, which are accumulated inside the endosomes prior its transport to the nucleus (Figure 5). Furthermore, cationic MSNs tend to be trapped within endosomes [42]. By contrast, DOX fluorescence from **CPT-PEG@MSN-DOX-GA** did not colocalize with endosomes (Figure 6i and Figure S9i) suggesting that a rapid endosome escape occurred. In that sense, it has been reported that negatively charged MSNs are prompt to escape from the endosome [31,42]. This assumption agrees with the anionic character of this nanoparticle (−20 mV).

### 3.6. Cytotoxicity

#### 3.6.1. Assessment of the Cytotoxic Effect of CPT vs. CPT-PEG in Combination with DOX toward HepG2 Cells

**Figure 7:**
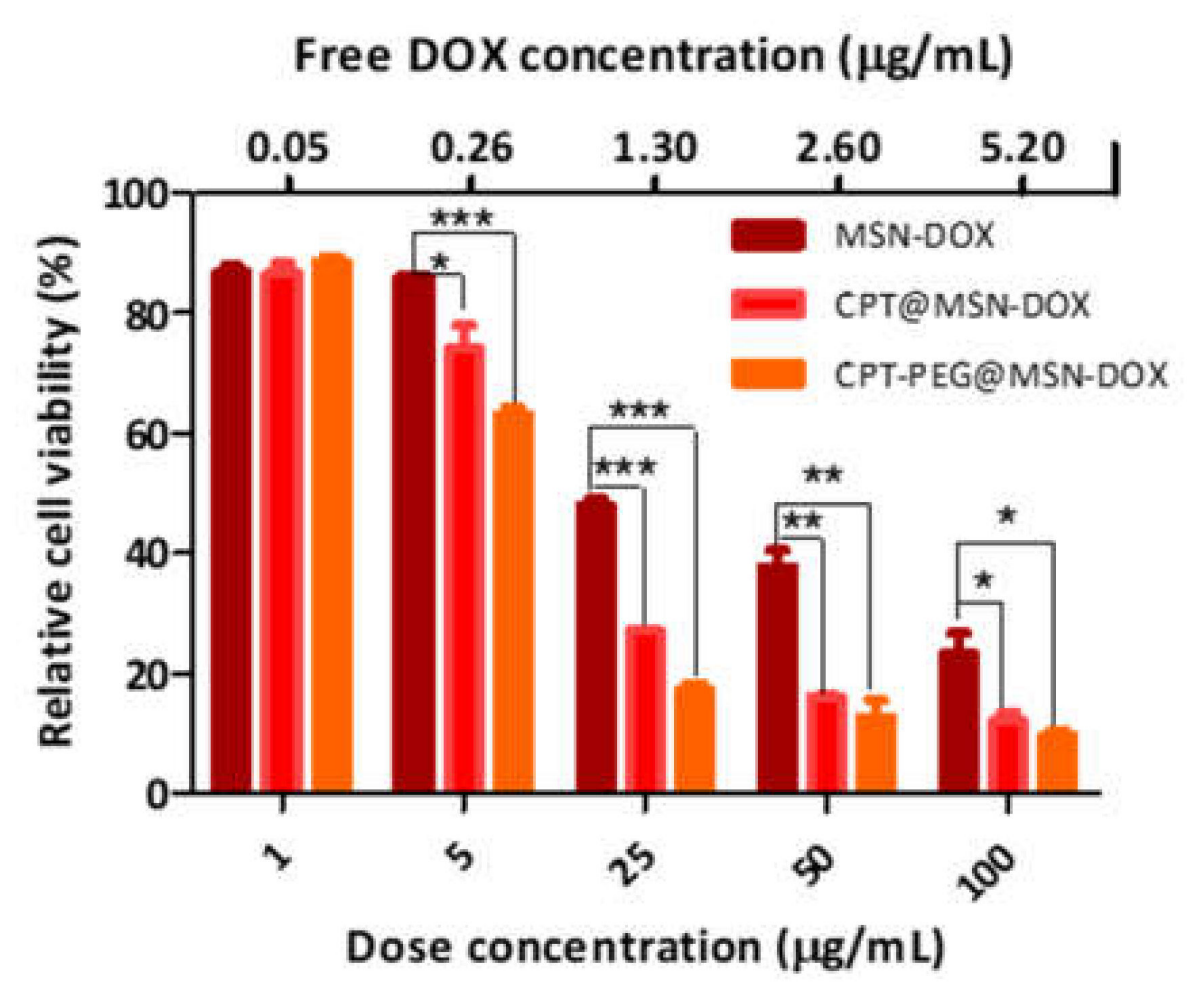
Cell viability of HepG2 incubated with **MSN-DOX**, **CPT@MSN-DOX**, and **CPT-PEG@MSN-DOX** for 72 h. Data represented as mean ± SD (*n* = 3), * *p* < 0.05, ** *p* < 0.01, and *** *p* < 0.0001. The concentration of free DOX is the released drug at pH 6.5. The concentration of free DOX was estimated as the released drug at pH 6.5 by **CPT-PEG@MSN-DOX**.

First, with the aim to assess the performance of the DDS containing prodrug CPT-PEG, the cytotoxicities of the nanoparticles loaded with CPT-PEG and DOX (**CPT-PEG@MSN-DOX**) were compared with the nano-vehicles containing the combination CPT/DOX and DOX (**CPT@MSN-DOX** and **MSN-DOX**). MTT cell viability assays were carried out with HepG2 cells for 72 h in concentrations ranging from 1 to 100 μg·mL^−1^ (Figure 7). The assays revealed a decrease in the relative cell viability at 5 μg·mL^−1^ from 85% (**MSN-DOX**) to 63% (**CPT-PEG@MSN-DOX**). At 25 μg·mL^−1^ of MSN, the increase in the cell death was found to be more pronounced. In presence of **CPT-PEG@MSN-DOX**, HepG2 cells viability dropped to 17%, whereas for **MSN-DOX**, the viability was 47%. These values are in line with reported results and confirm the synergistic activity of the combination of DOX/CPT [5,25].

Next, when nanoparticles containing CPT-PEG were compared with those loaded with CPT, it was found that there was an enhancement in the cytotoxic effect in the whole range of concentrations, especially from 5 to 50 μg·mL^−1^. For instance, **CPT-PEG@MSN-DOX** was about a 15% more cytotoxic than **CPT@MSN-DOX** at 5 μg·mL^−1^. At 25 μg·mL^−1^, the improvement was about 40%. This higher efficiency was translated into an improved synergistic activity. The calculated value of the combination index (CI) for the prodrug is 0.028, while system **CPT-PEG@MSN-DOX** displays a CI = 0.54.

#### 3.6.2. Cytotoxicity of **CPT-PEG@MSN-DOX-GA** toward HepG2 Cells

As demonstrated in the aforementioned study, the combination of CPT-PEG and DOX showed a high cytotoxicity in HepG2 cell line (Figure 7). It is expected that this toxicity would be similar for cell lines with no GA-receptors. To verify this premise, the toxicity of the same combination of drugs was examined using a similar DDS in HeLa cell line under identical experimental conditions. The results, as anticipated, showed that the toxicity found was indeed very high (Figure S10) reaching values of about 10% at concentration of nanoparticles of 25 μg·mL^−1^. In light of the high potency of this combination of drugs, a selective delivery of the drugs is needed. Therefore, the targeted nano-vehicle **CPT-PEG@MSN-DOX-GA** could minimize the off-target cytotoxicity while killing the Hepatocellular carcinoma cells.

**Figure 8:**
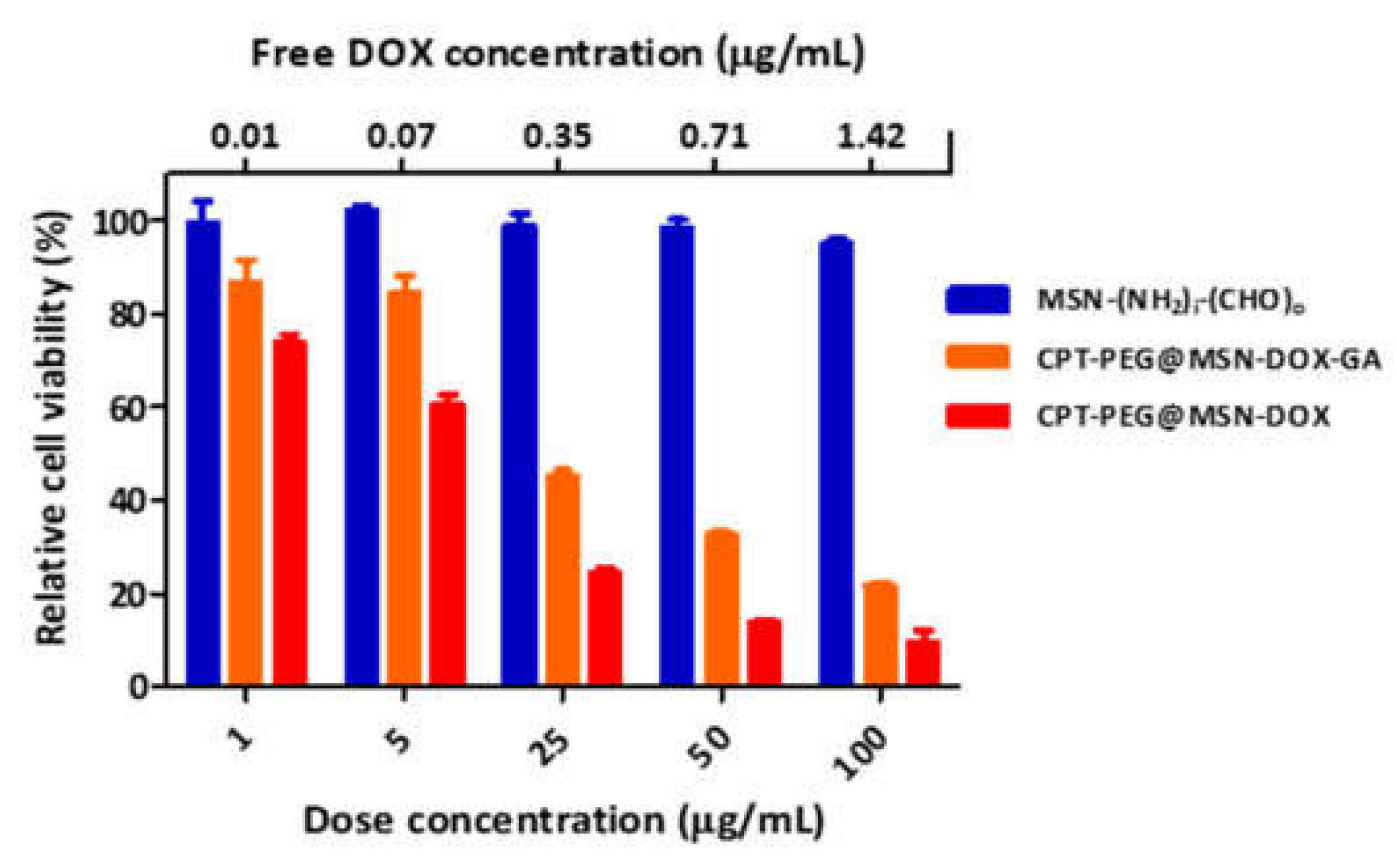
Cell viability of HepG2 cells incubated with **MSN-(NH_2_)_i_-(CHO)_o_**, **CPT-PEG@MSN-DOX-GA**, and **CPT-PEG@MSN-DOX** for 72 h. Data represented as mean ± SD (*n* = 3). The concentration of free DOX was estimated as the released drug at pH 6.5 by **CPT-PEG@MSN-DOX**.

To test the performance of this targeted DDS, the cytotoxicities of nanoparticles **CPT-PEG@MSN-DOX** and **CPT-PEG@MSN-DOX-GA** were determined by measuring the growth inhibition of HepG2 cells and compared. To do so, MTT assays with **MSN-(NH_2_)_i_-(CHO)_o_**, as control, **CPT-PEG@MSN-DOX** and **CPT-PEG@MSN-DOX-GA** were carried out (Figure 8). Gratifyingly, the results obtained showed an efficient cytotoxicity of the targeted system (**CPT-PEG@MSN-DOX-GA**). At a concentration of 100 μg·mL^−1^ of nanoparticle and an equivalent concentration of 1.42 μg·mL^−1^ of DOX, cell viability was about 20% [17]. A dose dependent trend is observed within the concentrations of nanoparticles tested. In contrast, **CPT-PEG@MSN-DOX-GA** displayed a modest cytotoxicity against HeLa cells, which is in agreement with the limited uptake of HeLa cells of such particles. (Figure 4b). The targeted DDS showed a lower toxicity compared to the non-targeted nanoparticles. This result was rationalized in terms of lower internalization of **CPT-PEG@MSN-DOX-GA** into the cells. As depicted in Figure 5, the relative uptake of the targeted DDS was about 20% lower after 24 h. As a consequence, lower doses of drugs were actually delivered to the cells.

Therefore, it is concluded that the decoration of the DDS with GA keeps an efficient cytotoxic activity, while endowing the nanoparticles with a high selectivity toward HepG2 cells (9 to 1, HepG2 vs. HeLa cells, Figure 4). With these features, it is foreseen that the present nanoparticles can contribute to design and implement efficacious treatments with a reduced systemic toxicity.

## 4. Conclusions

A dual Doxorubicin/Camptothecin-PEG pH-triggered drug delivery mesoporous silica nanoparticle-based nano-vehicle has been successfully prepared. The enhanced solubility of CPT-PEG in respect to that of CPT allows increasing the loading of the nanoparticles. In the presence of glutathione, CPT-PEG yields CPT in about 1 h. The substitution of CPT by CPT-PEG in combination with DOX results in an improved cytotoxicity of HepG2 cells in respect to the treatment with CPT/DOX.

With the aim to introduce selectivity toward HepG2 cells, the nanoparticles were decorated with glycyrrhetinic acid (**CPT-PEG@MSN-DOX-GA**). Uptake cytometry assays have demonstrated that these nanoparticles were selectively internalized by HepG2 cells. This selectivity suggests that the nanoparticles were internalized by a receptor–mediated endocytosis.

DOX released from **CPT-PEG@MSN-DOX-GA** was rapidly localized in the nuclei. This result was rationalized in terms of efficient endosomal escape, ascribed to the presence of the anionic charge of the glycyrrhetinic acid. The cytotoxicity of **CPT-PEG@MSN-DOX-GA** toward HepG2 cells have been assessed and are about 80% at doses of 100 µg/mL of the nanoparticles. Therefore, it is concluded that the presence of GA in **CPT-PEG@MSN-DOX-GA** can reduce the systemic toxicity of the combined treatment while still keeping an efficient cytotoxic activity.

Currently, the preparation of DDSs with an optimized internalization keeping their cellular selectivity is undertaken in our laboratory.

## Supplementary Materials

The following are available online at https://www.mdpi.com/1999-4923/12/11/1048/s1, Figure S1: ^1^H-NMR of (a) compound **2** and (b) CPT-PEG (**1**), Figure S2: ESI-FIA-TOF spectrum of CPT-PEG (**1**), Figure S3: Cleavage study of CPT-PEG with GSH 10 mM in PBS 7.4 for 1 h, Table S1: MSN-(NH_2_)_i_(CHO)_o_ size an ζ potential (pH = 5.5), Table S2: N_2_ adsorption-desorption and BJH pore size distribution values of MSN-(NH_2_)_i_(CHO)_o_, Figure S4: TEM micrographs of (a) MSN-(NH_2_)_i_(CHO)_o_ and (b) CPT-PEG@MSN-hyd-PEG-hyd-DOX, Scheme S1: Schematic synthesis of 3,6,9,12,15-pentaoxaheptadecanedihydrazide, Figure S5: ^1^H-RMN spectra of PEG-CHO (**5**) and its precursor, Figure S6: 1H-RMN comparison between GA-COOH (**6**) and GA-CHO (**3**), Figure S7: ESI-FIA-TOF-spectrum of GA-CHO (**3**), Figure S8: Zeta potential dependence of pH, Figure S9: CLSM images of the uptake of CPT-PEG@MSN-DOX and CPT-PEG@MSN-DOX-GA (100 μg·mL^−1^) at 37 °C for 10 h at 100 μg·mL^−1^. Scale bar 20 μm. Blue: DAPI, red: DOX, green: RAB7, Figure S10: Cell viability of HeLa cells incubated with MSN-(NH_2_)_i_-(CHO)_o_ and CPT-PEG@MSN-DOX for 72 h. Data represented as mean ± SD (*n* = 3), Equation (S1): Combination index (CI): ICx(A) and ICx(B) are the IC of drug A and drug B given individually and ICx(A)comb and ICx(B)comb are the IC values of the drugs combined to achieve the same viability inhibition (IC: inhibition concentration, x is the % of cell viability inhibition). Values of CI < 1 indicate synergism effect.
