# Supplementary material for: Glycyrrhetinic Acid-Functionalized Mesoporous Silica Nanoparticles for the Co-Delivery of DOX/CPT-PEG for Targeting HepG2 Cells
Source: Pharmaceutics. 2020 Nov 2;12(11):1048. doi: 10.3390/pharmaceutics12111048 (PMC7694026; doi:10.3390/pharmaceutics12111048)
Supplement: Supplementary file 1 [file pharmaceutics-12-01048-s001.pdf]

pharmaceutics

Supporting Information: Glycyrrhetinic acid functionalized Mesoporous Silica Nanoparticles for the co-delivery of DOX/CPT-PEG for targeting HepG2 cells.

Gabriel Martínez-Edo, Cristina Fornaguera, Salvador Borrós and David Sánchez-García *

1. Synthesis and characterization of CPT-PEG (1)

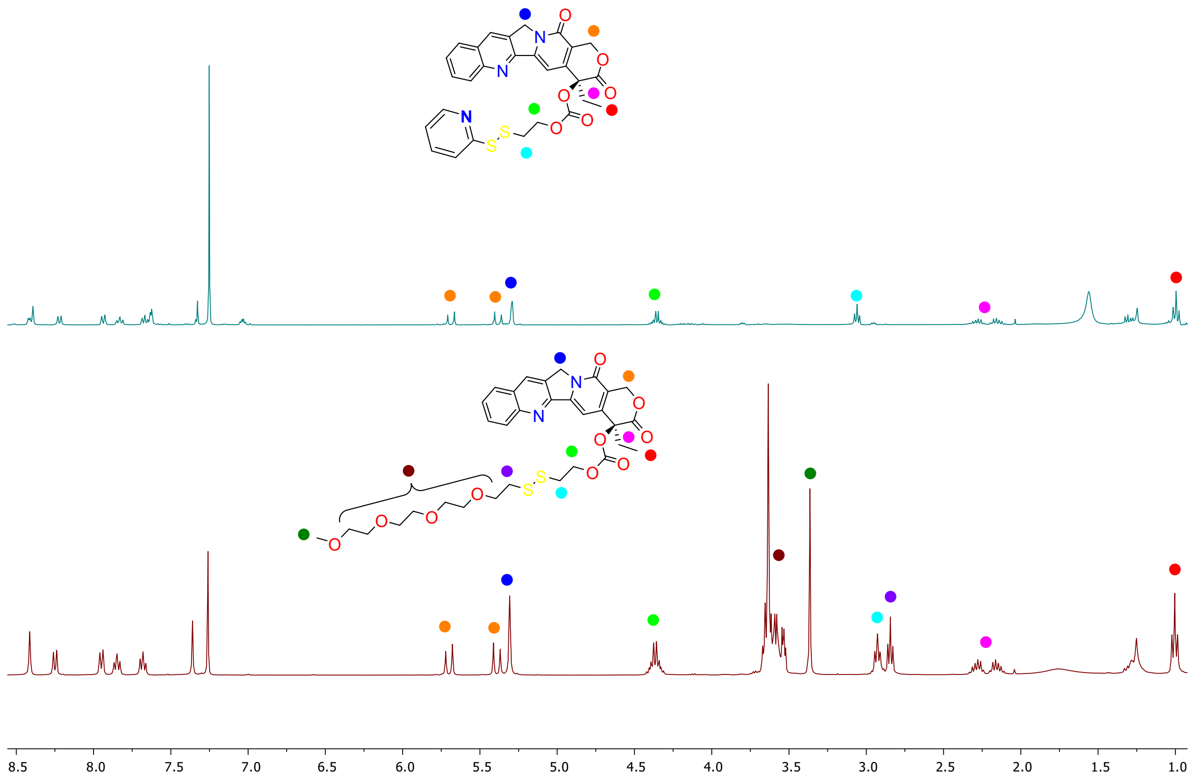

Figure S1. ^{1}H-NMR of a) compound **2** and b) CPT-PEG (**1**).

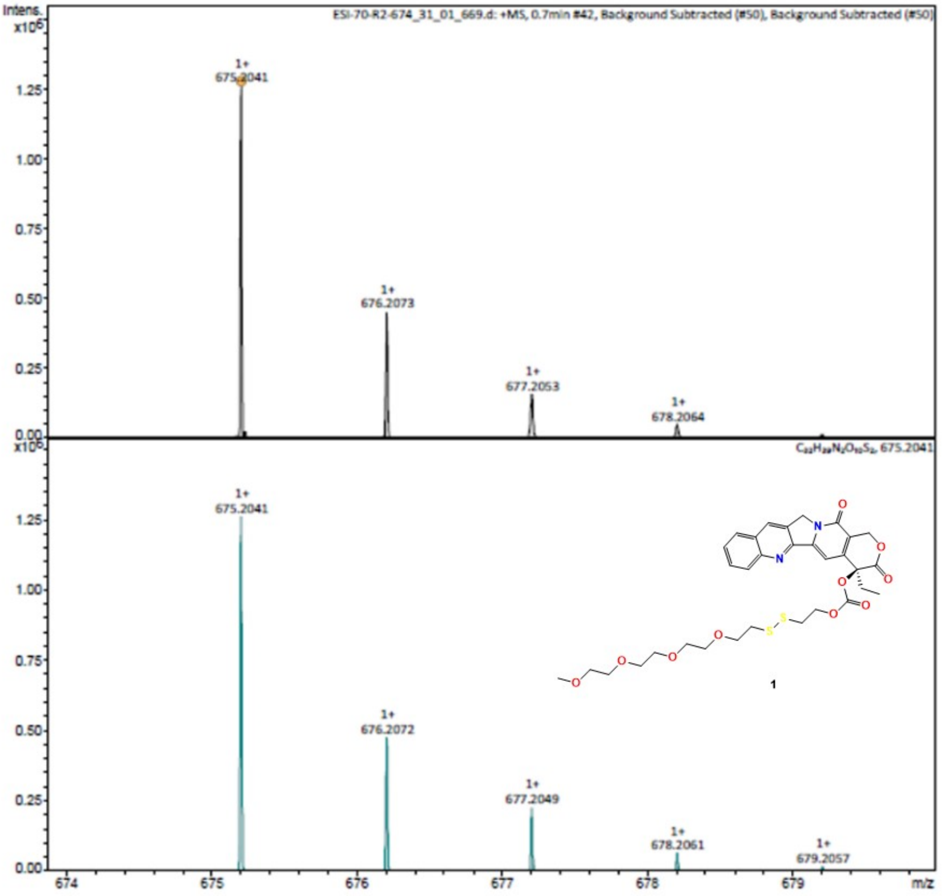

Figure S2. ESI-FIA-TOF spectrum of CPT-PEG (**1**).

2. CPT release from CPT-PEG

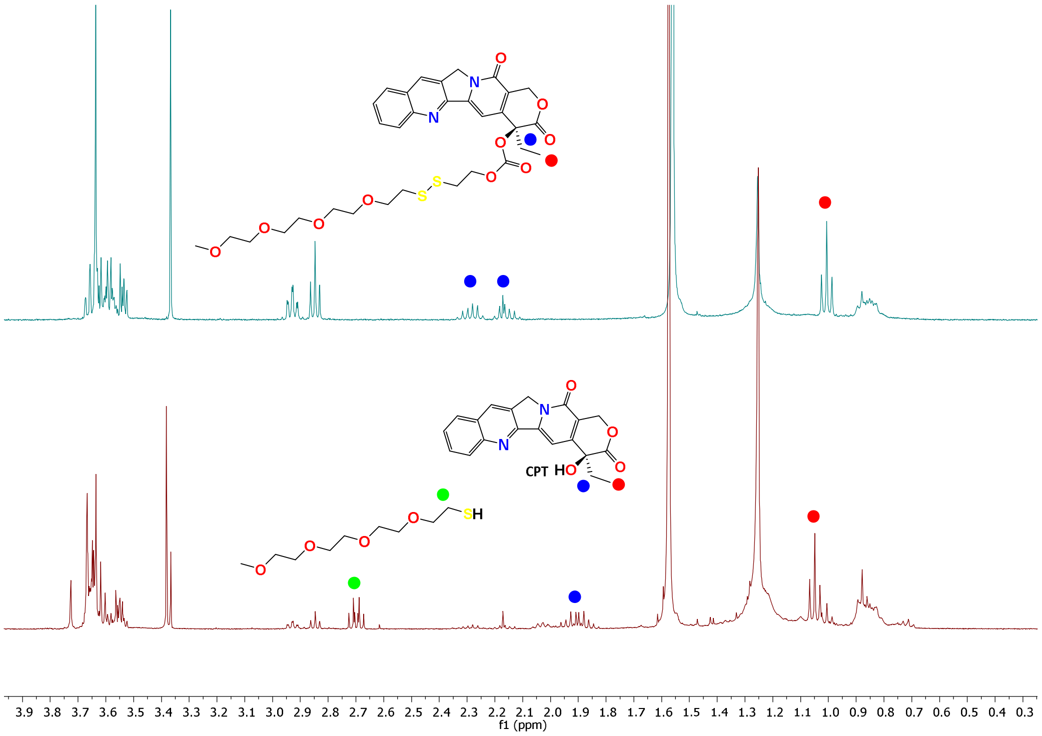

Figure S3. Cleavage study of CPT-PEG with GSH 10 mM in PBS 7.4 for 1 h.

3. Characterization of nanoparticles

Table S1. MSN-$(NH_2)_i(CHO)_o$ size an ζ potential (pH = 5.5).

**Size / nm**	**TEM**	**DLS**	**pdI**	**ζ-pot / mV**
MSN-(NH_2)	100	140 ± 4	0.04 ± 0.02	-1 ± 4
MSN-$(NH_2)_i(CHO)_o$	100	145 ± 5	0.17 ± 0.005	-14 ± 4
MSN-$(NH_2)_i$(hyd-PEG-hyd)$_o$	100	190 ± 5	0.06 ± 0.02	-10 ± 5

Table S2. N_2 adsorption-desorption and BJH pore size distribution values of MSN-$(NH_2)_i(CHO)_o$.

	**MSN-NH_2(CTAB)**	**MSN-(NH_2)**	**MSN-$(NH_2)_i(CHO)_o$**
BET surface area / $m^2 \cdot g^{-1}$	50 ± 0.2	1011 ± 10	990 ± 6
BJH pore volume / $cm^3 \cdot g^{-1}$	0.02 ± 0.01	0.66 ± 0.05	0.56 ± 0.1
Pore size / nm	--	2.4 ± 0.2	2.4 ± 0.2

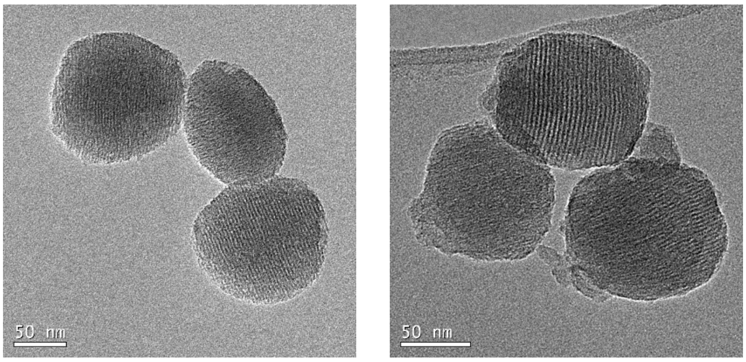

Figure S4. TEM micrographs of (a) MSN-$(NH_2)_i(CHO)_o$ and (c) CPT-PEG@MSN-hyd-PEG-hyd-DOX.

4. Synthesis of 3,6,9,12,15-pentaoxaheptadecanedihydrazide

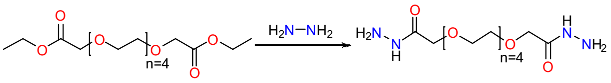

Scheme S1. Schematic synthesis of 3,6,9,12,15-pentaoxaheptadecanedihydrazide.

3,6,9,12,15-pentaoxaheptadecanedihydrazide 2 was synthetized following the procedure of Li et al. [1] Spectroscopic data were in good agreement with reported data. ^{1}H-NMR (400 MHz, $CDCl_3$): δ 6.81 (brs, 6H), 4.07 (s, 4H), 3.71-3.64 (m, 16H). ^{13}C-NMR (100 MHz, $CDCl_3$) δ 169.6, 77.0, 72.6, 70.1, 61.5.

5. Synthesis and characterization of GA-aldehyde (GA-CHO)

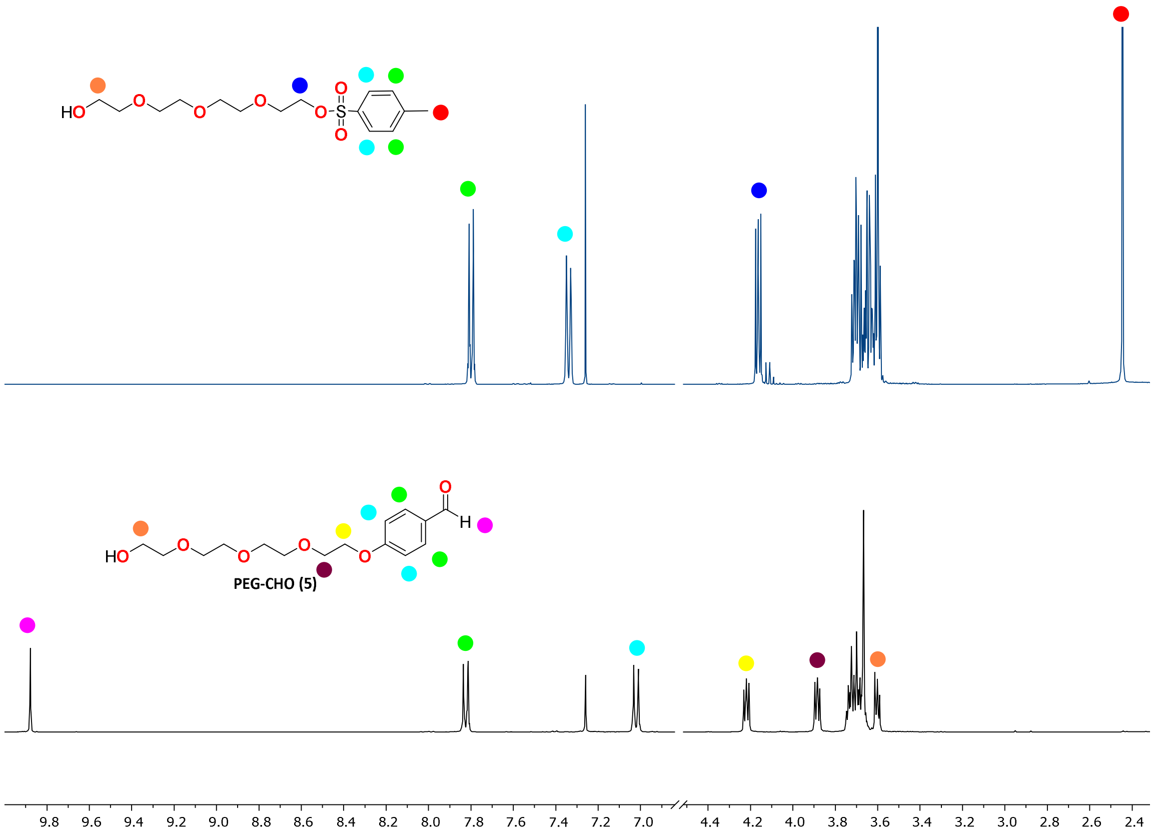

Figure S5. ^{1}H-RMN spectra of PEG-CHO (**5**) and its precursor.

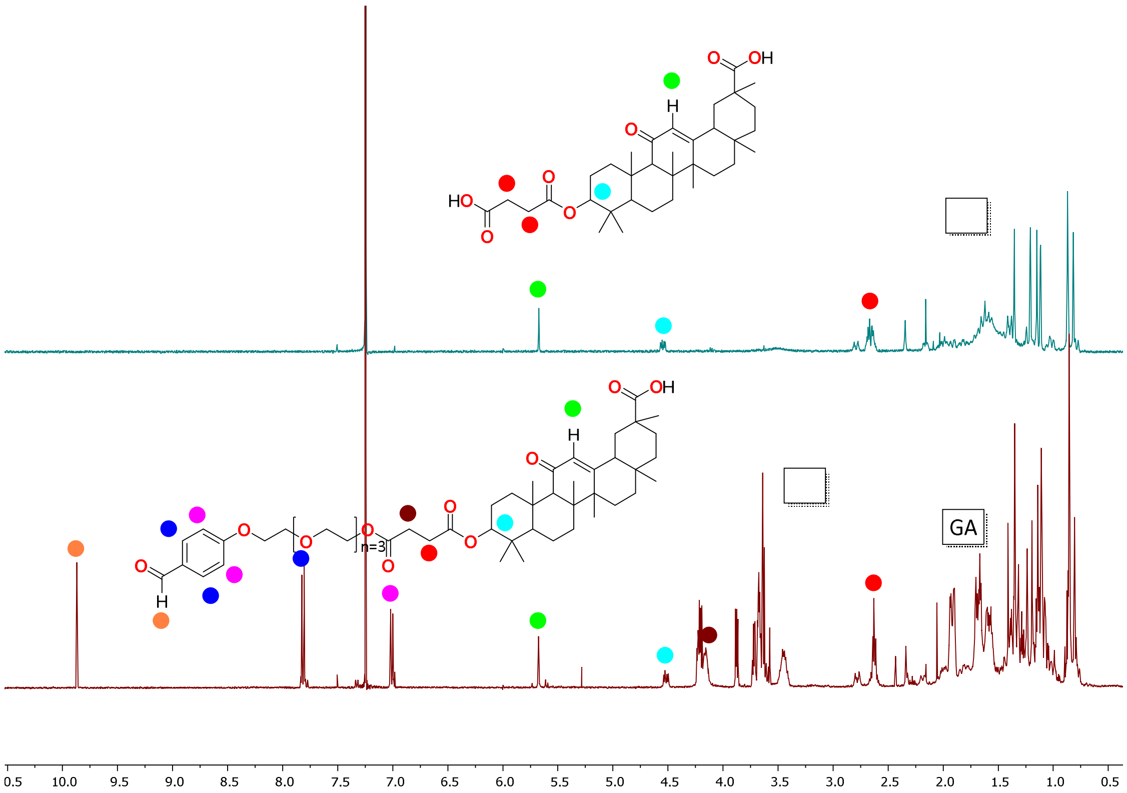

Figure S6. ^{1}H-RMN comparison between GA-COOH (**6**) and GA-CHO (**3**).

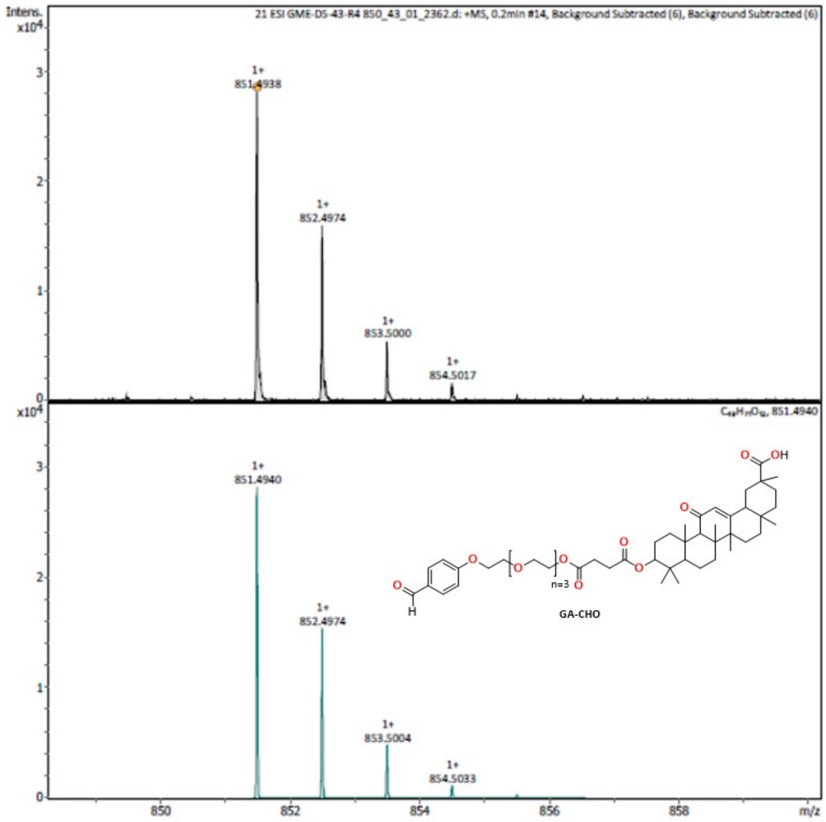

Figure S7. ESI-FIA-TOF-spectrum of GA-CHO (**3**).

6. CPT-PEG@MSN-DOX-GA Zeta potential dependence of pH

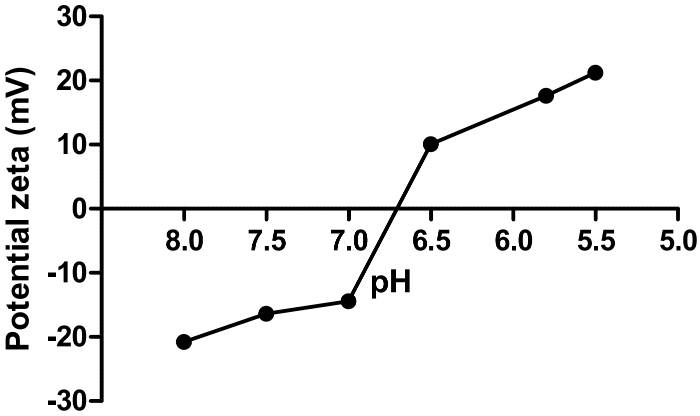

Figure S8. Zeta potential dependence of pH.

7. Confocal laser scanning microscopy (CLSM)

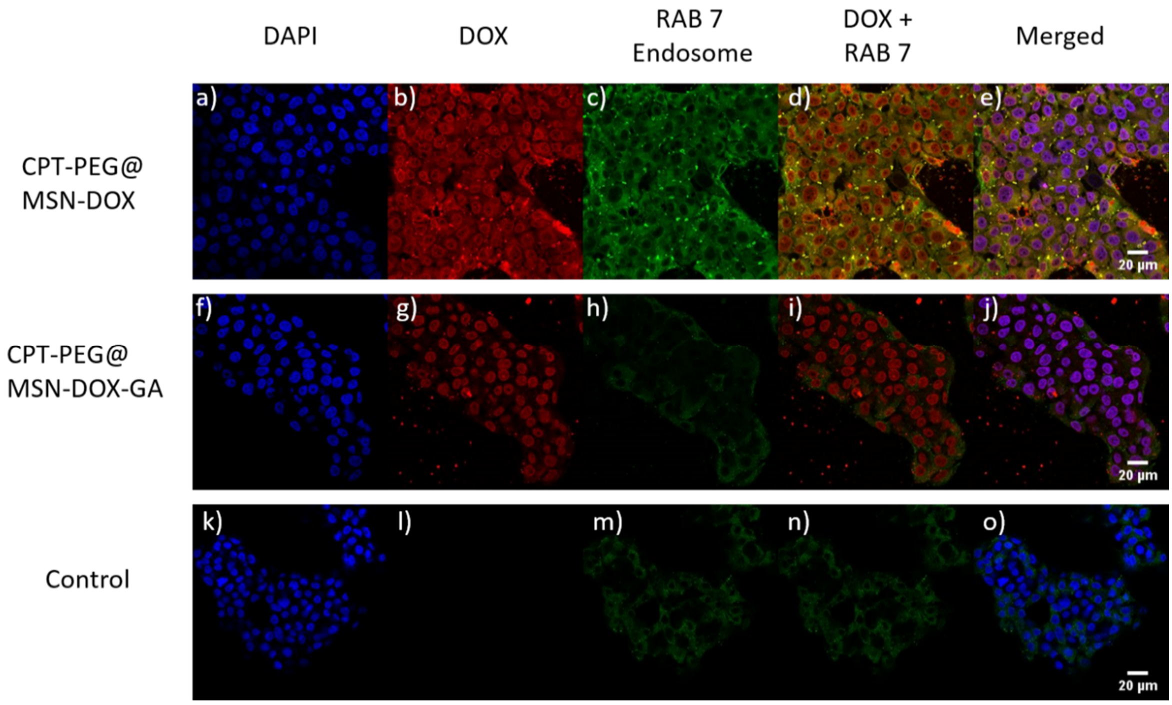

Figure S9. CLSM images of the uptake of **CPT-PEG@MSN-DOX** and **CPT-PEG@MSN-DOX-GA** (100 µg·mL^{-1}) at 37 °C for 10 h at 100 µg·mL^{-1}. Scale bar 20 µm. Blue: DAPI, red: DOX, green: RAB7.

8. Cytotoxicity of CPT-PEG@MSN-DOX toward HeLa cells

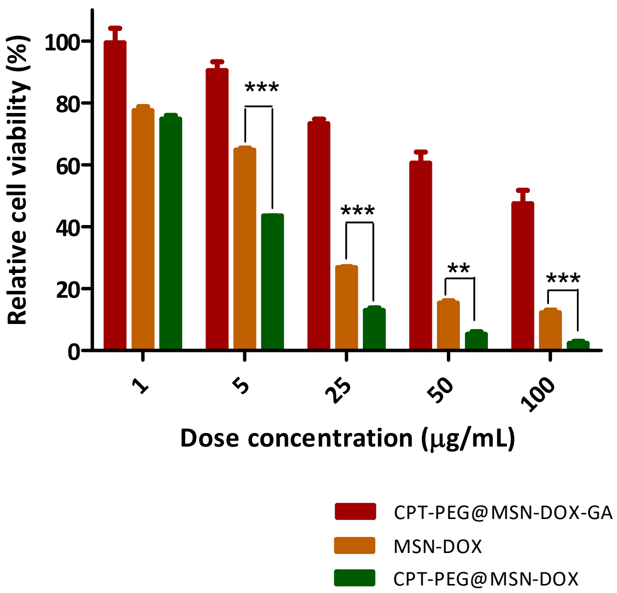

Figure S10. Cell viability of HeLa cells incubated with **CPT-PEG@MSN-DOX-GA, MSN-DOX** and **CPT-PEG@MSN-DOX** for 72 h. Data represented as mean ± SD (n = 3).

9. Combinational Index

$$CI = \frac{\mathrm{IC}_x(\mathrm{A})_{comb}}{\mathrm{IC}_x(\mathrm{A})} + \frac{\mathrm{IC}_x(\mathrm{B})_{comb}}{\mathrm{IC}_x(\mathrm{B})}$$

Equation S1. Combination index (CI): ICx(A) and ICx(B) are the IC of drug A and drug B given individually and $ICx(A)_{comb}$ and $ICx(B)_{comb}$ are the IC values of the drugs combined to achieve the same viability inhibition (IC: inhibition concentration, x is the % of cell viability inhibition). Values of CI <1 indicate synergism effect [2,3].

10. Bibliography

1. Li, Z.-Y.; Liu, Y.; Wang, X.-Q.; Liu, L.-H.; Hu, J.-J.; Luo, G.-F.; Chen, W.-H.; Rong, L.; Zhang, X.-Z. One-Pot Construction of Functional Mesoporous Silica Nanoparticles for the Tumor-Acidity-Activated Synergistic Chemotherapy of Glioblastoma. *ACS applied materials & interfaces* **2013**, *5*, 7995-8001, doi:10.1021/am402082d.
2. Li, Z.Y.; Liu, Y.; Wang, X.Q.; Liu, L.H.; Hu, J.J.; Luo, G.F.; Chen, W.H.; Rong, L.; Zhang, X.Z. One-pot construction of functional mesoporous silica nanoparticles for the tumor-acidity-activated synergistic chemotherapy of glioblastoma. *ACS Appl. Mater. Interfaces* **2013**, *5*, 7995-8001, doi:10.1021/am402082d.
3. Botella, P.; Abasolo, I.; Fernández, Y.; Muniesa, C.; Miranda, S.; Quesada, M.; Ruiz, J.; Schwartz Jr, S.; Corma, A. Surface-modified silica nanoparticles for tumor-targeted delivery of camptothecin and its biological evaluation. *Journal of Controlled Release* **2011**, *156*, 246-257, doi:http://dx.doi.org/10.1016/j.jconrel.2011.06.039.
